# Supplementary material for: Metal-responsive regulation of enzyme catalysis using genetically encoded chemical switches
Source: Nat Commun. 2022 Apr 6;13:1864. doi: 10.1038/s41467-022-29239-y (PMC8987029; doi:10.1038/s41467-022-29239-y)
Supplement: Supplementary file 1 — Supplementary Information [file 41467_2022_29239_MOESM1_ESM.pdf]

SUPPORTING INFORMATION

Metal-Responsive Regulation of Enzyme Catalysis using Genetically Encoded Chemical Switches

Yasmine S. Zubi,[‡#] Kosuke Seki,[†#] Ying Li,[§] Andrew Hunt,[†] Bingqing Liu,[‡] Benoît Roux,[§*] Michael C. Jewett,[†*] Jared C. Lewis[‡*]

[#]These authors contributed equally to this study
**To whom correspondence should be addressed*

Table of Contents

Supplemental Figures 3

Synthetic Procedures 22

General Materials and Methods 22

Synthesis of BpyAla 22

Computational Procedures 27

Force Field Parameterization 27

MD Simulations of POP 30

MD Simulations of Luciferase 30

BpyAla Site Selection in POP 32

BpyAla Site Selection in Luciferase 33

High-Throughput Procedures and Screening 34

General Materials and Methods 34

DNA Cloning and Preparation Method 34

BpyRS purification 34

Cell extract preparation 35

Cell-free protein synthesis (CFPS) 35

POP Purification for MS Analysis 36

Intact Protein Mass Spectrometry 36

Protein Quantification in CFPS by Scintillation Counting 37

Autoradiogram analysis 37

High-Throughput Screening and Analysis of POP mutants 37

High-throughput screening of luciferase mutants 39

Kinetic analysis of luciferase mutants 40

Low-Throughput Procedures and Large-Scale Characterization 40

General Materials and Methods 40

Cloning of TAG (amber stop codon) POP variants 41

Large-Scale Protein Expression 42

Steady-State Kinetic Assays 43

Activity Switching Assays 44

Physical Characterization of POP Variants 45

References 55

Supplemental Figures

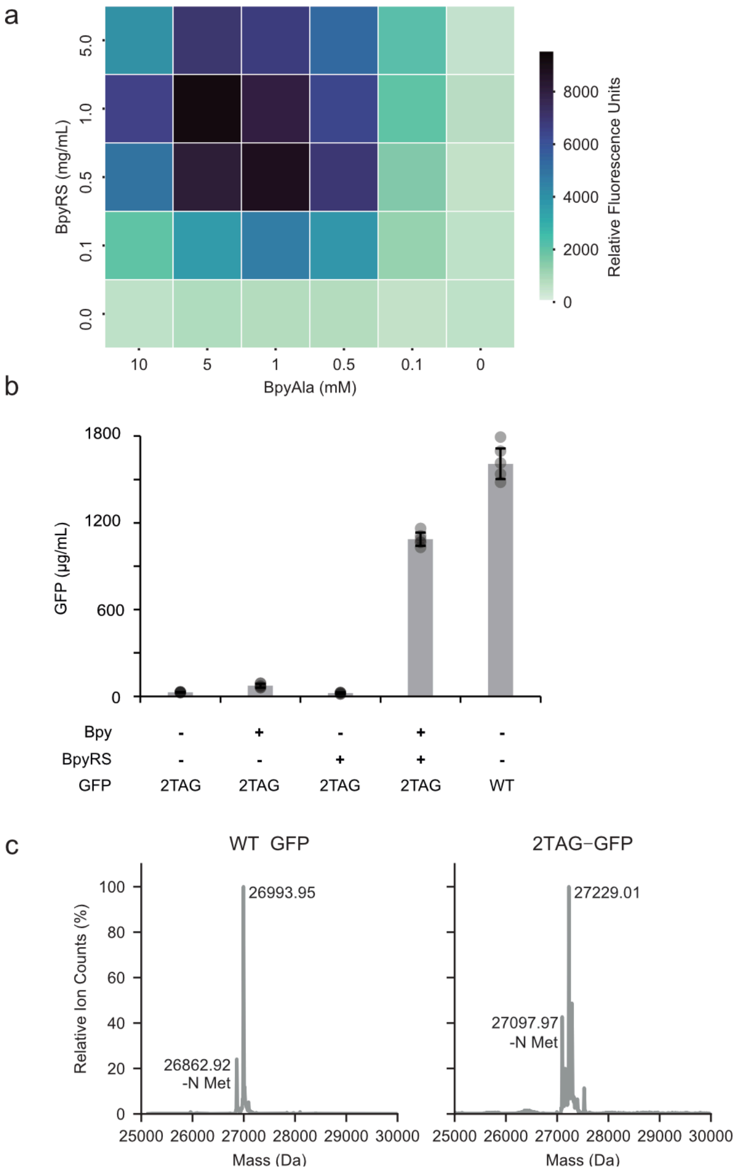

Supplementary Figure 1. BpyAla incorporation into sfGFP.
Cell-free protein synthesis (CFPS) can be optimized for efficient and accurate incorporation of a pair of BpyAla residues. **(a)** BpyAla and BpyRS concentrations are combinatorially mixed in CFPS reactions to synthesize 2TAG-sfGFP. 5 mM BpyAla and 1 mg/mL BpyRS is nearly equivalent to expression with 1 mM BpyAla and 0.5 mg/mL BpyRS, so the latter condition was used to conserve BpyAla and BpyRS. **(b)** Both BpyAla and BpyRS are required to synthesize active 2TAG-sfGFP. Translation in the presence of either one, or neither, component results in negligible amounts of active sfGFP. sfGFP yields are calculated using a standard curve generated by radioactive ^{14}C-leucine incorporation to convert RFU to mass (µg/mL). **(c)** Intact protein MS shows masses in good agreement with incorporation of both BpyAla residues. We observe truncation of the N-terminal methionine, which is a common post-translational modification in *E. coli.* Theoretical masses for WT sfGFP, WT sfGFP -N Met, 2TAG-sfGFP, and 2TAG-sfGFP -N Met are 26995.39, 26864.19, 27230.72, and 28099.53 Da. Data represent mean ± standard deviation from n=3 independent experiments.

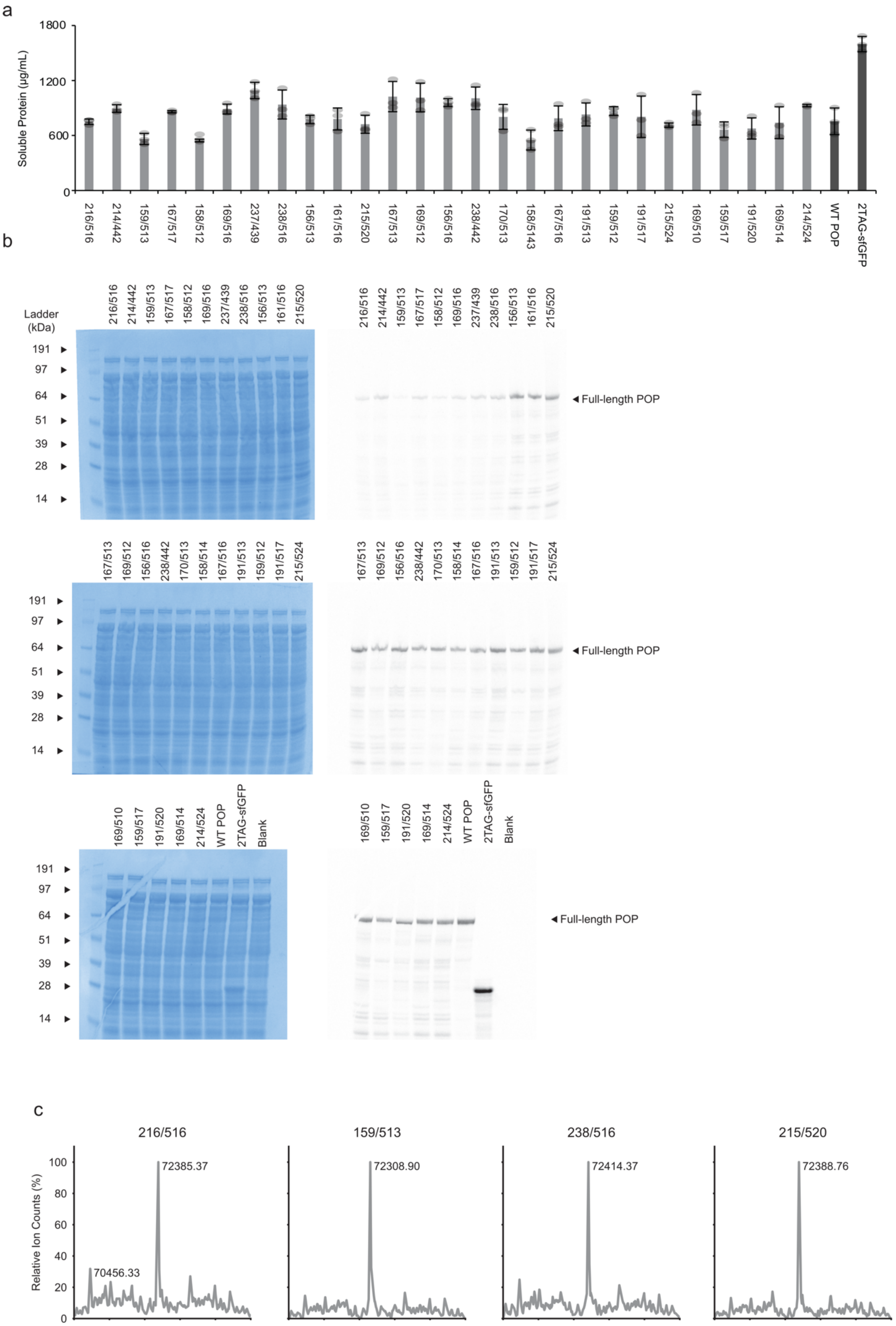
a
Soluble Protein (μg/mL)
1800
1200
600
0
216/516
214/442
159/513
167/517
158/512
169/516
237/439
238/516
156/513
161/516
215/520
167/513
169/512
156/516
238/442
170/513
158/5143
167/516
191/513
159/512
191/517
215/524
169/510
159/517
191/520
169/514
214/524
WT POP
2TAG-sfGFP
b
Ladder (kDa)
191
97
64
51
39
28
14
Full-length POP
Blank
c
216/516
159/513
238/516
215/520
Relative Ion Counts (%)
100
80
60
40
20
0
72385.37
70456.33
72308.90
72414.37
72388.76

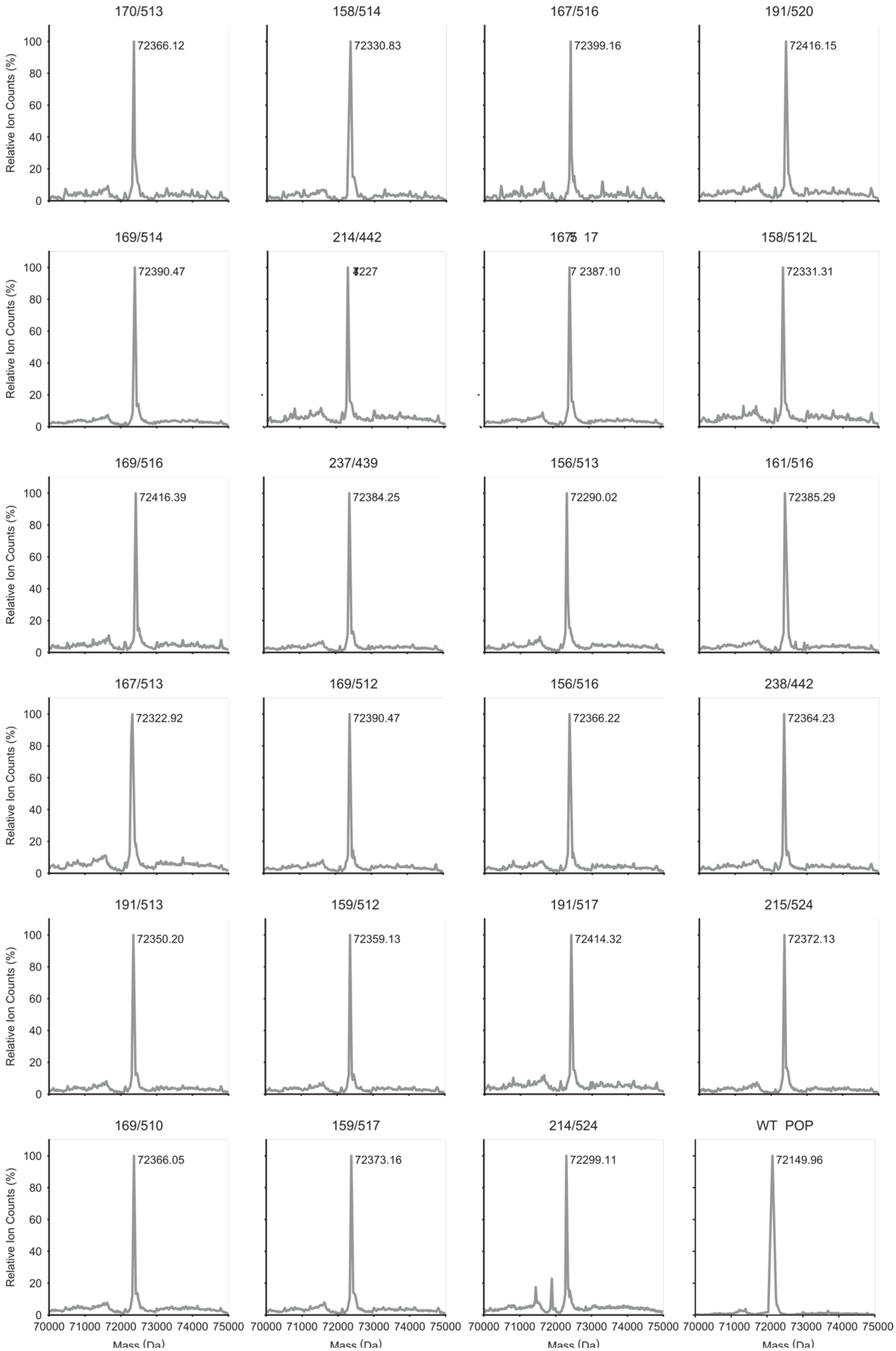
170/513
72366.12
158/514
72330.83
167/516
72399.16
191/520
72416.15
169/514
72390.47
214/442
7 2387.10
158/512L
72331.31
169/516
72416.39
237/439
72384.25
156/513
72290.02
161/516
72385.29
167/513
72322.92
169/512
72390.47
156/516
72366.22
238/442
72364.23
191/513
72350.20
159/512
72359.13
191/517
72414.32
215/524
72372.13
169/510
72366.05
159/517
72373.16
214/524
72299.11
WT POP
72149.96
Relative Ion Counts (%)
Mass (Da)
0
20
40
60
80
100
70000
71000
72000
73000
74000
75000

Supplementary Figure 2. BpyAla incorporation into POP.
A pair of BpyAla residues can be efficiently and accurately incorporated into all POP variants in CFPS. **(a)** All POP variants express solubly at yields comparable to WT POP, as measured by scintillation counting of proteins containing ^{14}C-leucine. Data represent mean $\pm$ standard deviation from n=3 replicates. **(b)** Analysis of POP mobility in SDS-PAGE and in autoradiograms shows major products of identical size as WT POP. Bands further down on the gel may indicate the presence of smaller products from protease cleavage, but no major truncation product bands were observed. **(c)** Intact protein MS analysis of purified POP proteins shows masses in good agreement with incorporation of a pair of BpyAla residues. Theoretical masses are shown in Supplementary Table 5. Data in b are representative of n=2 independent experiments, and data in c were collected once.

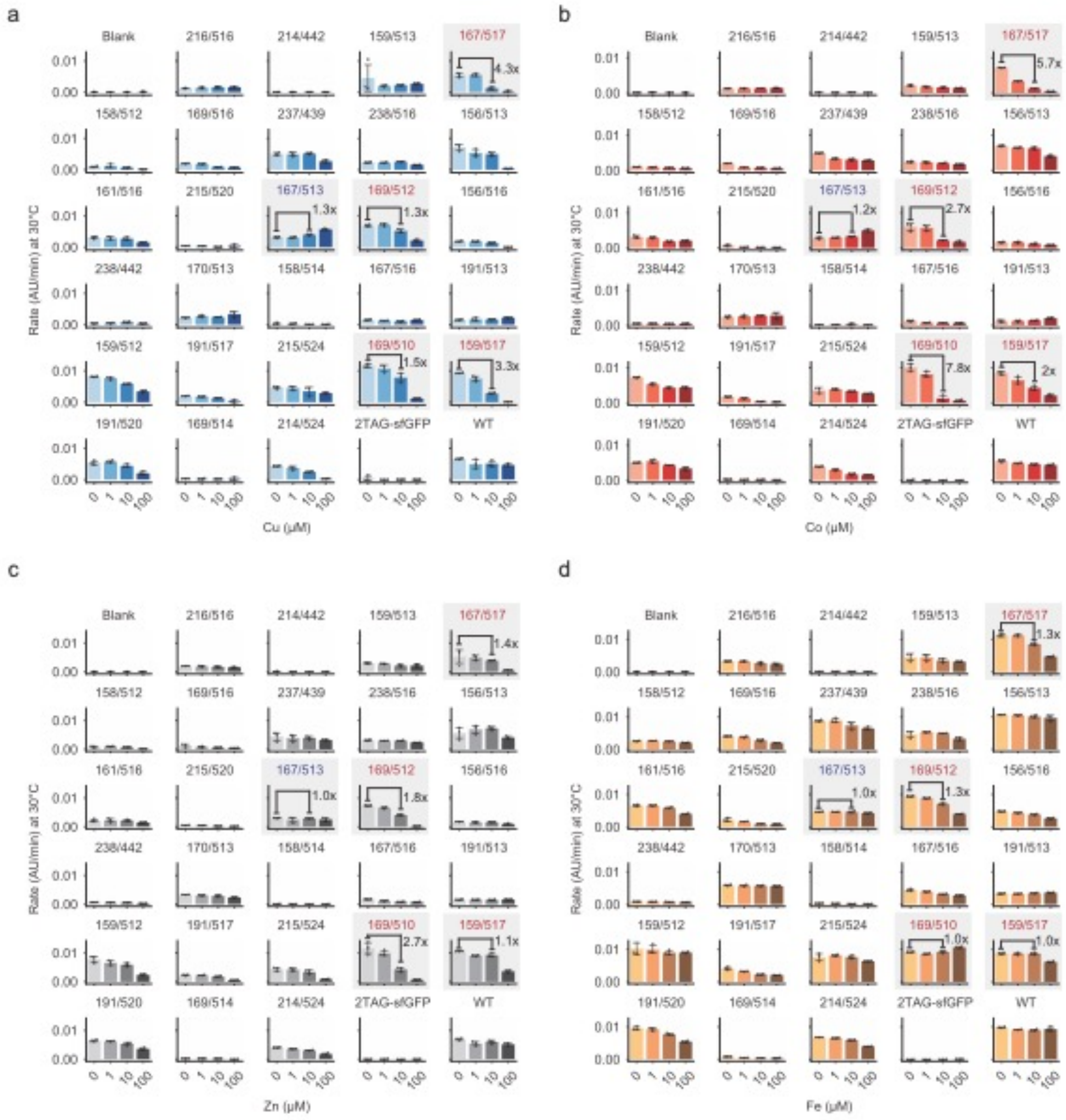

Supplementary Figure 3. High-throughput screening of POP activity in response to different divalent metals.

High-throughput experiments for POP rates in response to a range of **(a)** Cu, **(b)** Co, **(c)** Zn, and **(d)** Fe concentrations show that M(II) ions can inhibit and activate select POP variants in a dose dependent manner. Data represent mean ± standard deviation from n=3 replicates and were replicated in two independent experiments.

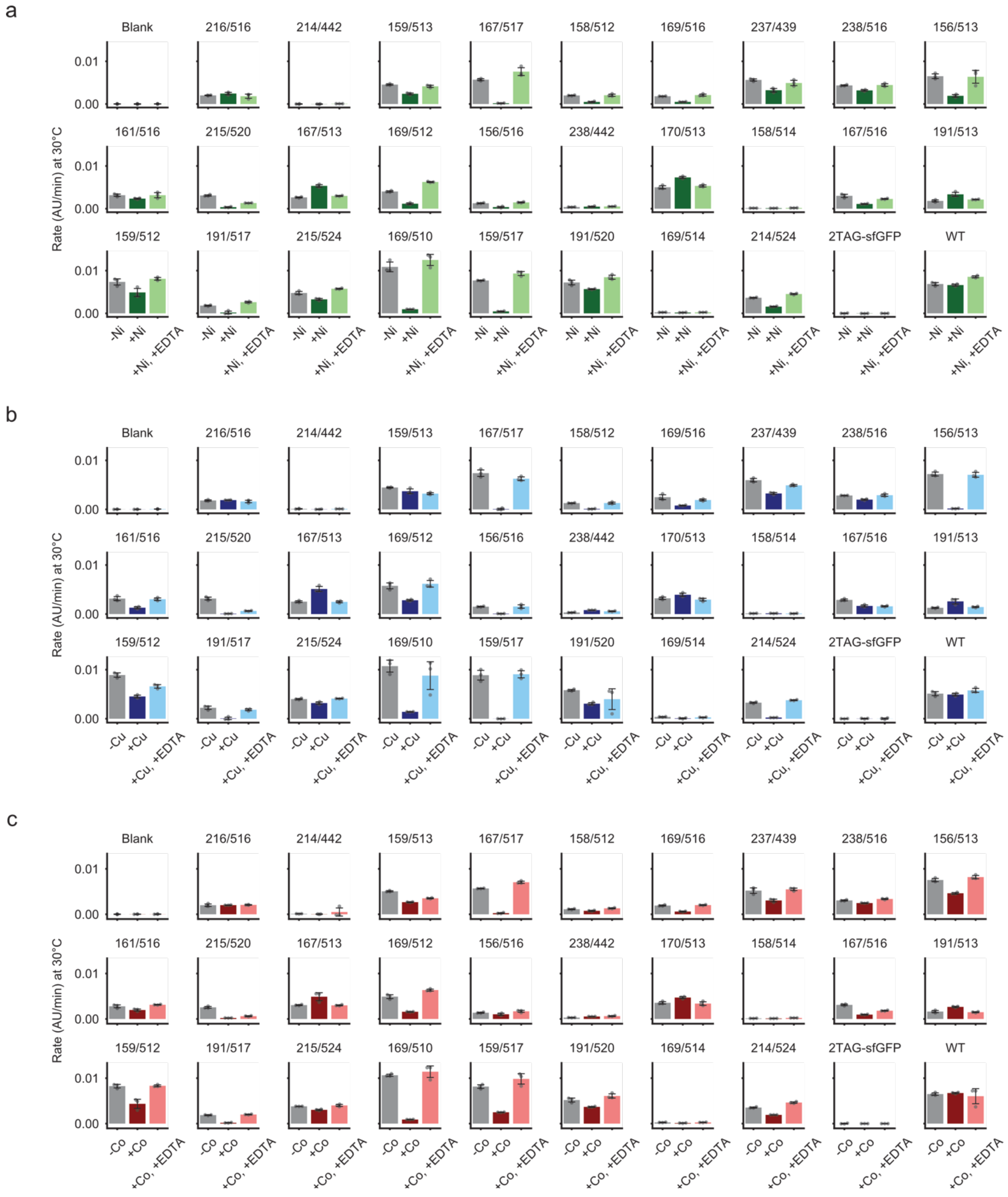
a
Blank
216/516
214/442
159/513
167/517
158/512
169/516
237/439
238/516
156/513
161/516
215/520
167/513
169/512
156/516
238/442
170/513
158/514
167/516
191/513
159/512
191/517
215/524
169/510
159/517
191/520
169/514
214/524
2TAG-sfGFP
WT
Rate (AU/min) at 30°C
0.01
0.00
-Ni
+Ni
+Ni, +EDTA
b
-Cu
+Cu
+Cu, +EDTA
c
-Co
+Co
+Co, +EDTA

d

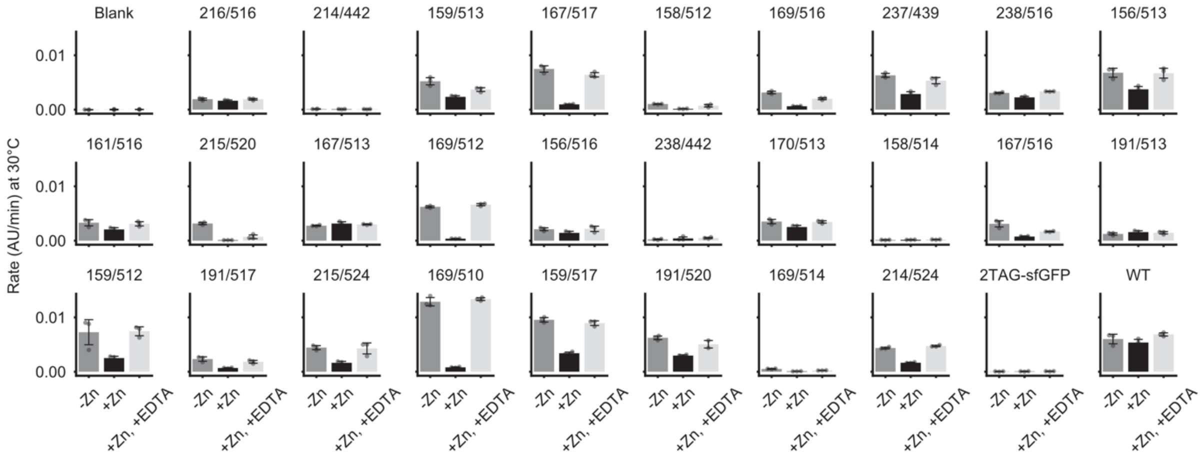

e

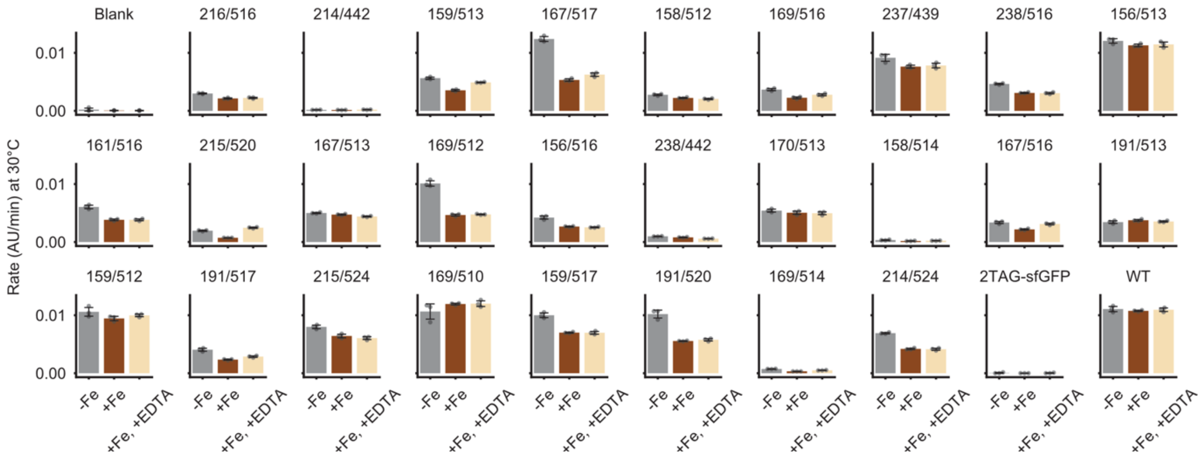

Supplementary Figure 4. High-throughput screening of reversibility of metal-dependent activity changes in POP.

High-throughput screening of reversibility shows that addition of EDTA to POP enzymes that are pre-treated with **(a)** Ni, **(b)** Cu, **(c)** Co, **(d)** Zn, and **(e)** Fe can restore apo-enzyme activity. Data represent mean ± standard deviation from n=3 independent replicates and were replicated in two independent experiments.

Supplementary Table 1. Residues chosen for mutation to BpyAla in Pfu POP.

Residue #	Residue *i*	Residue #	Residue *j*	Distance *i-j* Closed Conformation (Å)	Delta Open-Closed (Å)	Ni^{2+} Responsive[a]?	Active[b] ?
216	GLN	516	SER	11.0	7.3	No	No
158	ARG	512	LEU	9.9	6.1	Inhibited	No
156	PHE	513	TYR	11.1	6.1	Inhibited	Yes
169	PRO	512	LEU	11.1	6.0	Inhibited	Yes
170	ALA	513	TYR	11.3	5.9	Activated	No
191	SER	513	TYR	10.9	5.5	Activated	No
215	ASN	524	ASN	11.6	4.4	No	Yes
214	TRP	442	HSE	11.1	-3.2	Inhibited	Complete loss[c]
169	PRO	516	SER	9.8	-2.6	Inhibited	No
167	ASN	513	TYR	12.0	2.5	No	No
156	PHE	516	SER	11.9	2.3	Inhibited	No
158	ARG	514	ILE	9.7	-2.2	No	Complete loss[c]
159	LYS	512	LEU	11.5	1.8	No	Yes
169	PRO	510	HSE	12.5	1.8	Inhibited	Yes
169	PRO	514	ILE	10.6	-1.7	Inhibited	Complete loss[c]
159	LYS	513	TYR	10.1	-1.2	No	No
237	SER	439	GLU	11.8	-0.8	No	Yes
215	ASN	520	PRO	9.7	-0.7	No	Complete loss[c]
238	VAL	442	HSE	11.3	-0.6	No	Complete loss[c]
238	VAL	516	SER	11.6	10.6	No	No
167	ASN	516	SER	7.6	10	No	No
191	SER	520	GLU	10.7	9.2	No	Yes
167	ASN	517	VAL	11.2	10.7	Inhibited	Yes
161	LYS	516	SER	12.3	8.7	No	No
191	SER	517	VAL	9.7	9.1	Inhibited	No
159	LYS	517	VAL	10.5	9.5	Inhibited	Yes
214	TRP	524	ASN	11.5	4.2	Inhibited	Yes

[a] Defined as > 50% inhibition or activation at the highest concentration of metal tested compared to apo activity. [b] Defined as having > 50% WT activity [c] Complete loss indicates < 10% WT activity

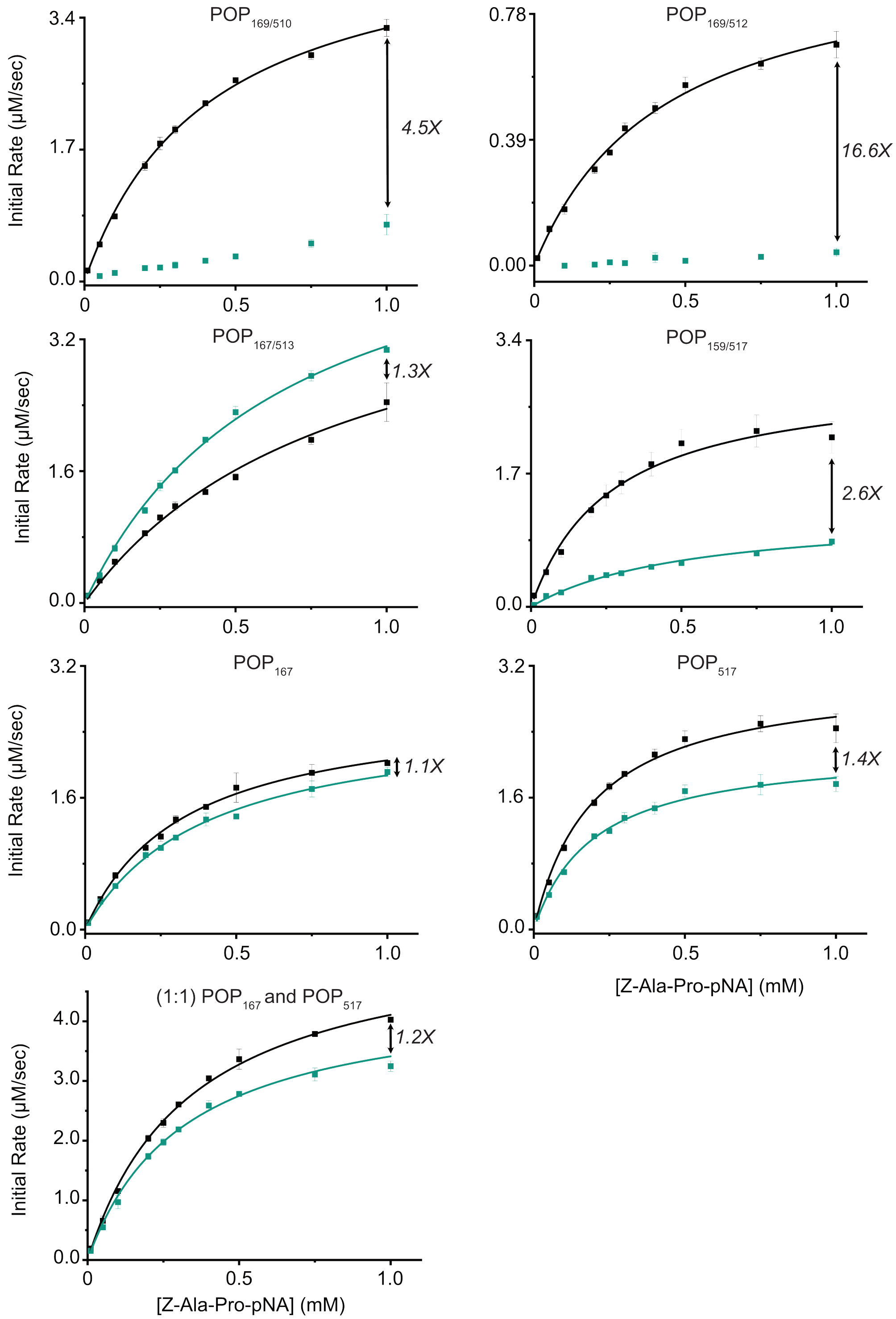

Supplementary Figure 5. Kinetic traces for BpyAla POP variants inhibited by Ni(II).
Steady-state kinetic assays were performed at 85 °C with purified enzymes (~10-30 nM) in either the presence of 1 mM EDTA (black) or 5 μM $NiCl_2$ (green). Initial rates of reaction (μM/sec) are plotted versus substrate concentration (mM) and data was fit, when appropriate, with the Michaelis-Menten equation (Equation 2). The fold-change (EDTA vs. Ni^{2+}) between reaction rates at 1 mM substrate is shown. Each data point represents the average of 3 replicates (n=3) and error bars represent standard deviations.

Supplementary Table 2. Steady-state kinetic parameters for selected POP variants.

	K_M (µM)		k_{cat} (sec^{-1})		V_{max} (uM/sec)[b]	
[a]***Variant***	***+ EDTA***	***+ NiCl₂***	***+ EDTA***	***+ NiCl₂***	***+ EDTA***	***+ NiCl₂***
$POP_{159/517}$	265 ± 38	523 ± 73	139 ± 8	57 ± 4	2.95 ± 0.16	1.21 ± 0.09
$POP_{169/510}$	409 ± 20	NA	154 ± 4	NA	4.60 ± 0.11	NA
$POP_{169/512}$	428 ± 36	NA	91 ± 4	NA	0.993 ± 0.039	NA
$POP_{167/513}$	854 ± 102	661 ± 43	219 ± 16	259 ± 9	4.37 ± 0.31	5.18 ± 0.18
(1:1) POP_{167} and POP_{517}	340 ± 19	317 ± 22	275 ± 7	225 ± 7	5.50 ± 0.13	4.49 ± 0.15

[a]*Reactions were conducted in triplicate using 0-1 mM Z-Ala-Pro-pNA and 20-21 nM enzyme in 10% v/v DMSO/30 mM HEPES (pH 7.4) containing 0.8 M NaCl at 85 °C for 1 minute. Rates were determined by changes in absorbance over time at 410 nm using a calculated molar extinction coefficient for pNA (7,126 M^{-1} cm^{-1}). Kinetic parameters were determined by the non-linear regression function in OriginPro using the Michaelis-Menten equation (Equation 2).* [b]V_{max} *is also provided due to the variable purity of the variants specified used here (see Supplementary Figure 6 below).*

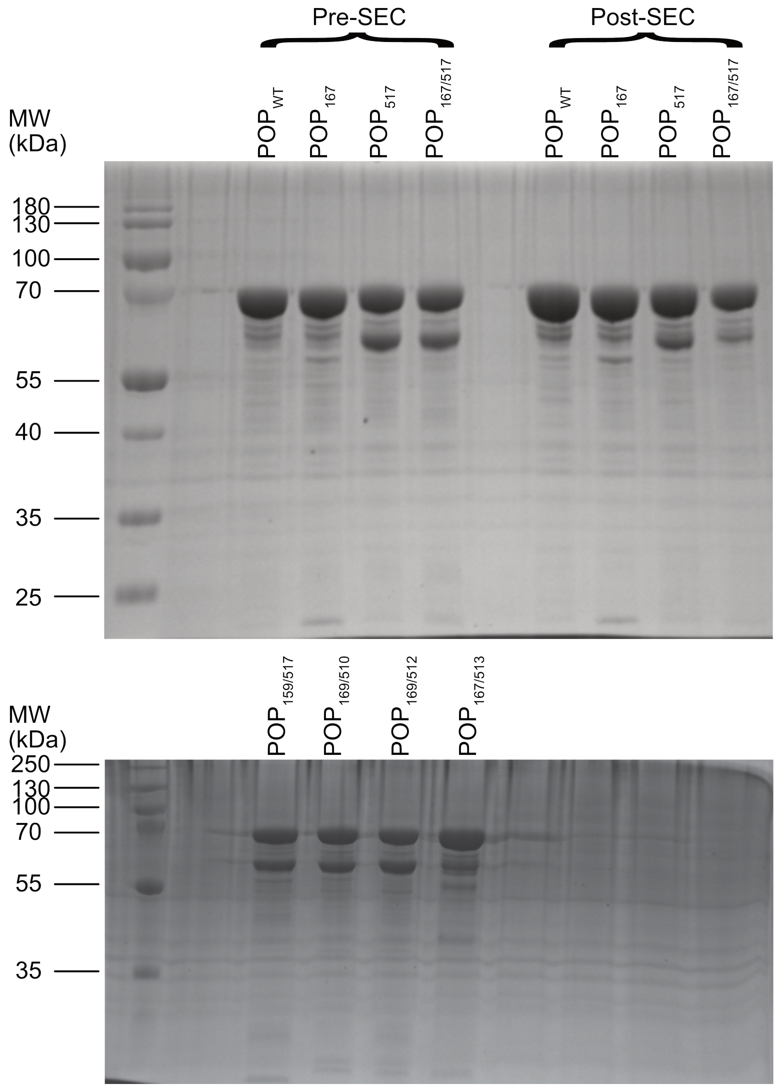

Supplementary Figure 6. Representative SDS-PAGE gel of purified POP variants.

SDS-PAGE analysis of (top) POP_{WT}, POP_{167}, POP_{517}, and $POP_{167/517}$ before and after SEC purification (SEC purification was performed in the presence of excess EDTA) and (below) $POP_{159/517}$, $POP_{169/510}$, $POP_{169/512}$, and $POP_{167/513}$ following IMAC purification. PageRuler™ Prestained Protein Ladder or PageRuler™ Plus Prestained Protein Ladder (Thermo Fisher) were used for reference. 4 µg of each protein sample was loaded onto the gel. In addition to a band corresponding to the MW of the desired full-length protein, we also observe varying amounts of the truncated protein depending on the variant (<5% - ~40%), that forms because of competition between release factor 1 mediated (RF1) termination of translation and BpyAla incorporation at the amber stop codon[1]. Truncated protein was isolated along with full-length protein after IMAC due to apparent affinity of the truncated POP side products for Ni-NTA. The MW of the variants and relevant truncated forms are as follows: POP_{WT} (71,888.78 Da); POP_{167} full-length (71,999.93 Da); POP_{167} truncated (19405.80 Da); POP_{517} full-length (72,014.90 Da); POP_{517} truncated (59,306.42 Da); $POP_{167/517}$ full-length (72,126.04 Da); $POP_{167/517}$ 517-truncated (59,330.50 Da); $POP_{159/517}$ full-length (72,111.98 Da); POP_{159} truncated (18,550.84 Da); $POP_{159/517}$ 517-truncated (59,403.50 Da); $POP_{169/510}$ full-length (72,105.03 Da); POP_{169} truncated (19,617.02 Da); $POP_{169/510}$ 510-truncated (58,635.63 Da); $POP_{169/512}$ full-length (72,129.02 Da); $POP_{169/512}$ 512-truncated (58,900.95 Da); $POP_{167/513}$ full-length (72,062.01 Da); $POP_{167/513}$ 513-truncated (58,997.11 Da).

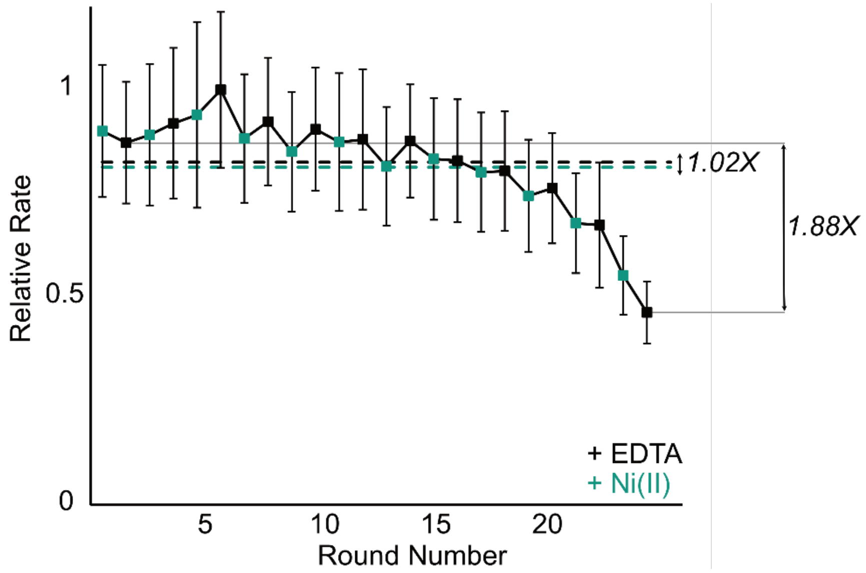

Supplementary Figure 7. Switching assay with POP$_{WT}$.

Switching kinetic assays were performed at 85 °C with purified enzyme (20 nM) after each iterative addition of either EDTA (black) or $NiCl_2$ (green). Relative rates of reaction are plotted versus round number. The average rates in the presence of excess EDTA or Ni(II) over the entirety of the assay are depicted by black and green dashed lines, respectively. There is a 1.02-fold change between these average rates. Additionally, the change in relative rate from the first addition of EDTA (round 2) and the final addition of EDTA (round 24) is shown (1.88-fold decrease). Reactions were conducted in triplicate using 1 mM Z-Ala-Pro-*p*NA in 10% v/v DMSO/30 mM HEPES (pH 7.4) containing 0.1 M NaCl at 85 °C for 1 minute. Rates were determined by changes in absorbance over time at 410 nm using a calculated molar extinction coefficient for *p*NA (7,126 M^{-1} cm^{-1}). Data points are averages of triplicate reactions (n=3) and error bars represent standard deviations.

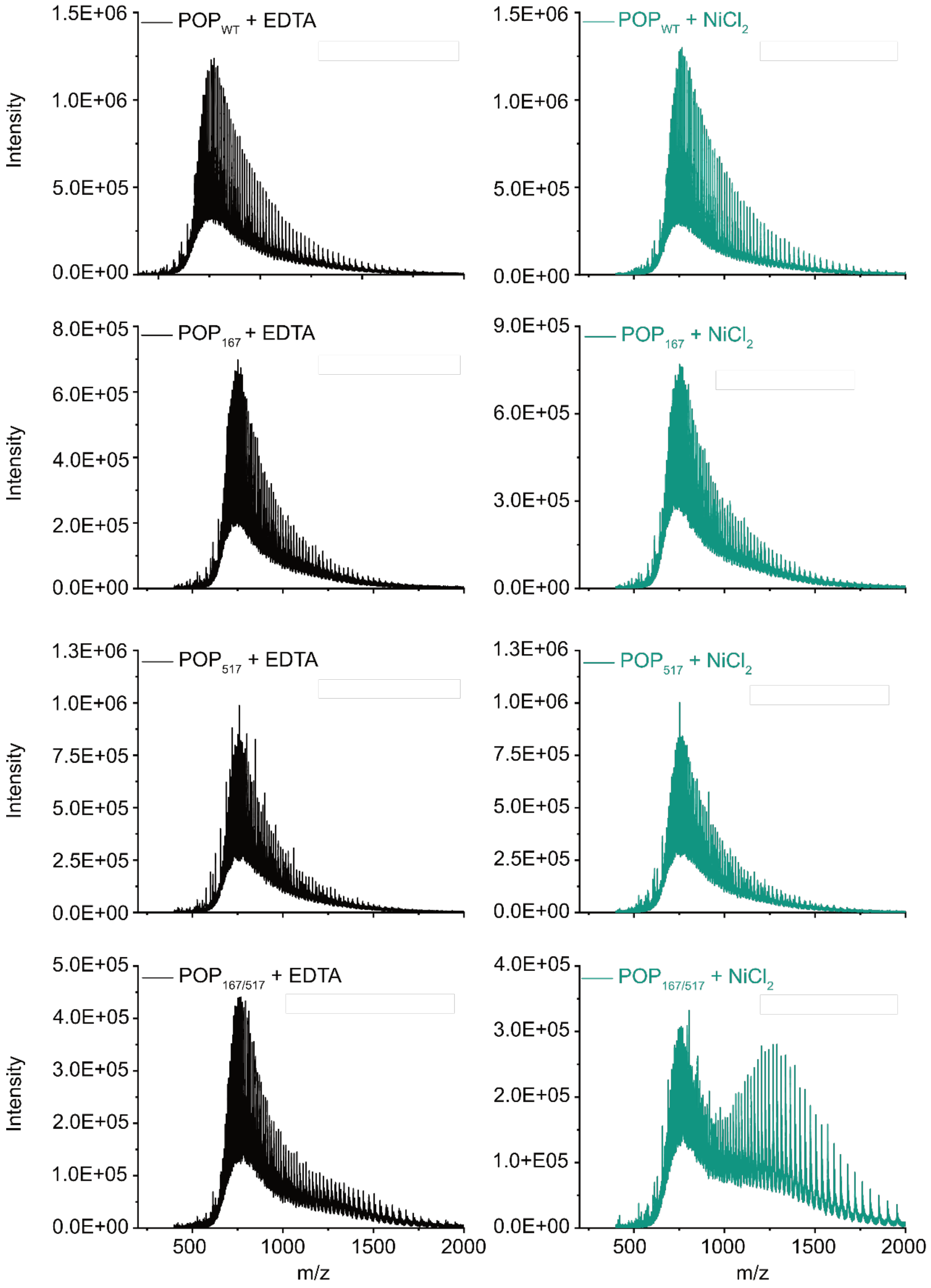

Supplementary Figure 8. Raw MS of POP variants treated with EDTA or NiCl$_2$.
Raw MS data (n=1) for POP$_{WT}$, POP$_{167}$, POP$_{517}$, and POP$_{167/517}$ in the presence of excess EDTA (black) or Ni^{2+} (green).

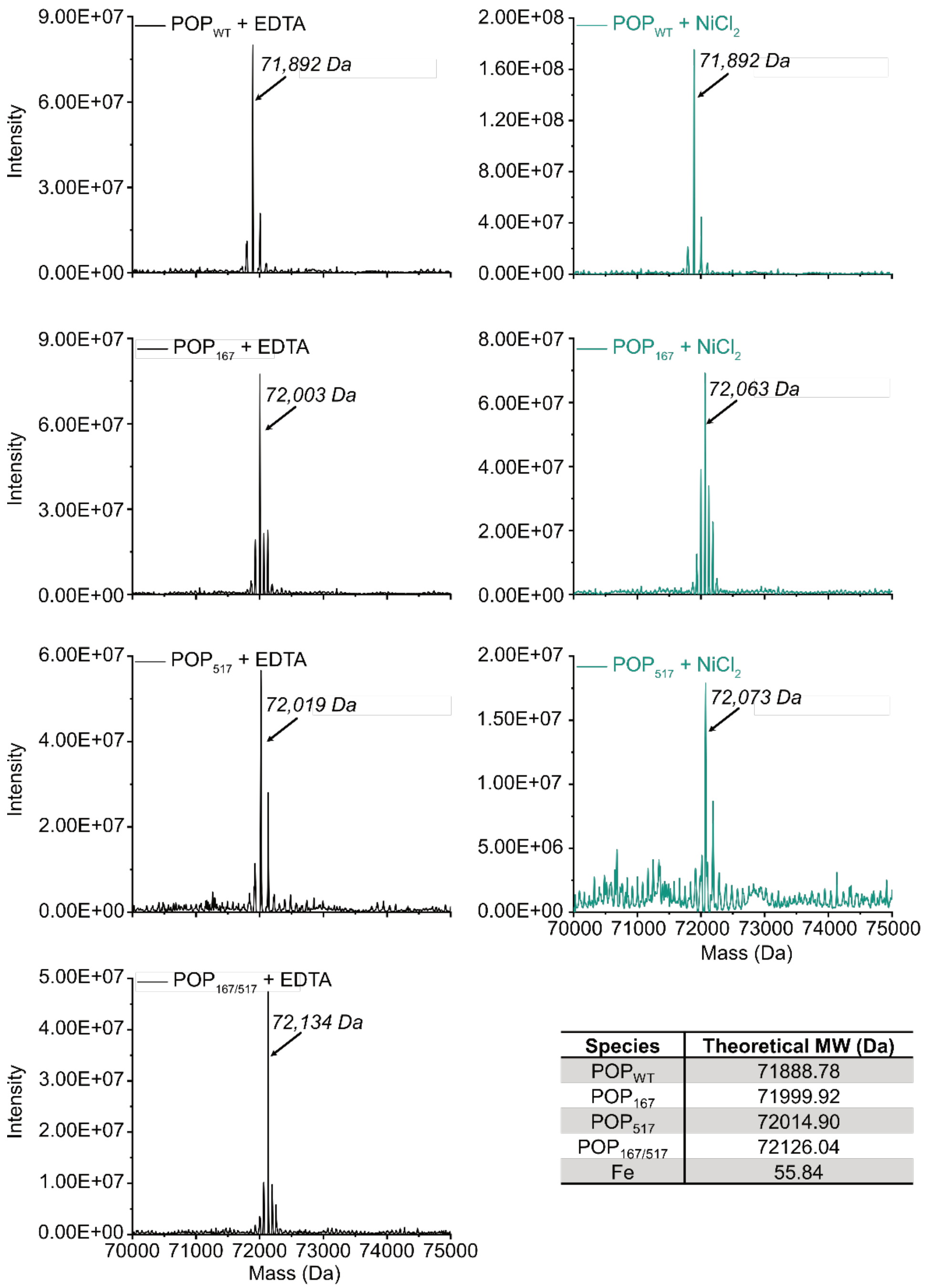

Species	Theoretical MW (Da)
POP$_{WT}$	71888.78
POP$_{167}$	71999.92
POP$_{517}$	72014.90
POP$_{167/517}$	72126.04
Fe	55.84

Supplementary Figure 9. MS data for POP variants treated with EDTA or NiCl$_2$.
Deconvoluted MS data (n=1) for POP$_{WT}$, POP$_{167}$, POP$_{517}$, and POP$_{167/517}$ in the presence of excess EDTA (black) or NiCl$_2$ (green). Data was deconvoluted using a 700-900 m/z window and the masses of the most intense from each sample are highlighted. Additionally, the expected MW of the apo enzymes is shown in a table in the bottom right of the figure. POP$_{WT}$ MS had the most intense mass corresponding to the apo protein in both the EDTA and Ni(II) samples. For all BpyAla variants, deconvoluted spectra revealed apo masses. For POP$_{167}$ and POP$_{517}$, single metalation was suggested by deconvoluted spectra. Deconvoluted MS for Ni(II)-treated POP$_{167/517}$ can be found in main text Figure 4c.

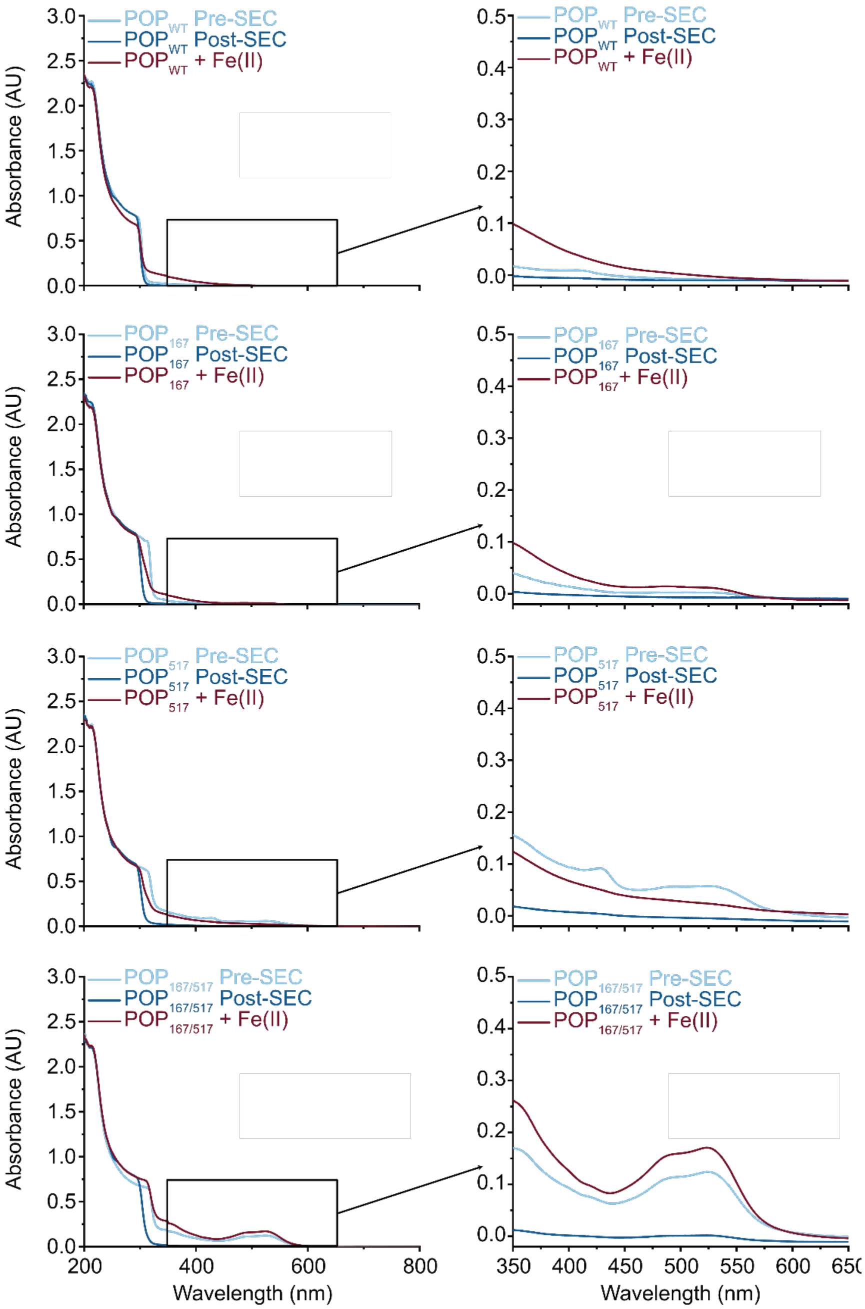

Supplementary Figure 10. UV-Vis spectra of various POP samples.
UV-Vis spectra were collected of POP_{WT}, POP_{167}, POP_{517}, and $POP_{167/517}$ (all at 50 μM) during the purification process. Protein samples were analyzed before treatment with 1,10-phenathroline (light blue) and then after treatment with the chelator and subsequent SEC purification (dark blue). Spectra were also collected after addition of Fe^{2+} (crimson red). Prior to treatment with phenanthroline, POP_{517} and $POP_{167/517}$ were pink, and peaks were observed in corresponding spectra between 450 and 600 nm (a zoomed in view of this region is shown on the right), which is consistent with an MLCT transition of an $Fe(BpyAla)_n$ complex[2]. After treatment with chelating agent, this feature disappeared in POP_{517} and $POP_{167/517}$. When treated with Fe(II), $POP_{167/517}$ regained its original pink color and the characteristic transition in the visible light region returned.

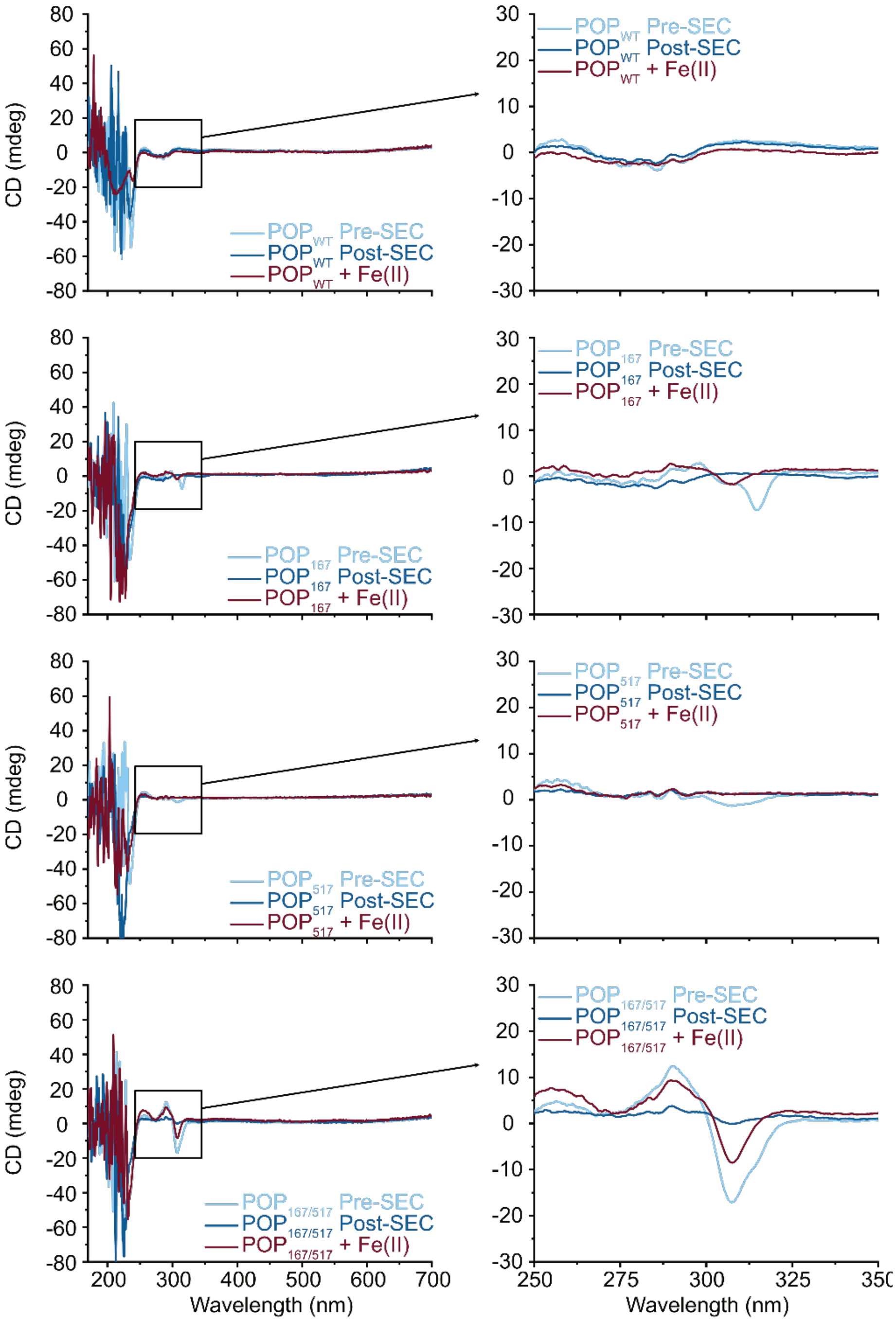

Supplementary Figure 11. CD spectra of various concentrated POP samples.

CD spectra were collected of POP_{WT}, POP_{167}, POP_{517}, and $POP_{167/517}$ (all at 50 μM) during the purification process. Protein samples were analyzed before treatment with 1,10-phenathroline (light blue) and then after treatment with the chelator and subsequent SEC purification (dark blue). Spectra were also collected after addition of Fe^{2+} (crimson red). Prior to treatment with phenanthroline, peaks were observed at around 280-325 nm for POP_{167}, POP_{517}, and $POP_{167/517}$ (a zoomed in view of this region is shown on the right), which is consistent with the expected intraligand charge transfer of BpyAla[3]. These features disappeared after treatment with chelating agent. When treated with Fe(II), only $POP_{167/517}$ regained its original pink color and the aforementioned Cotton effects in the near-UV region returned.

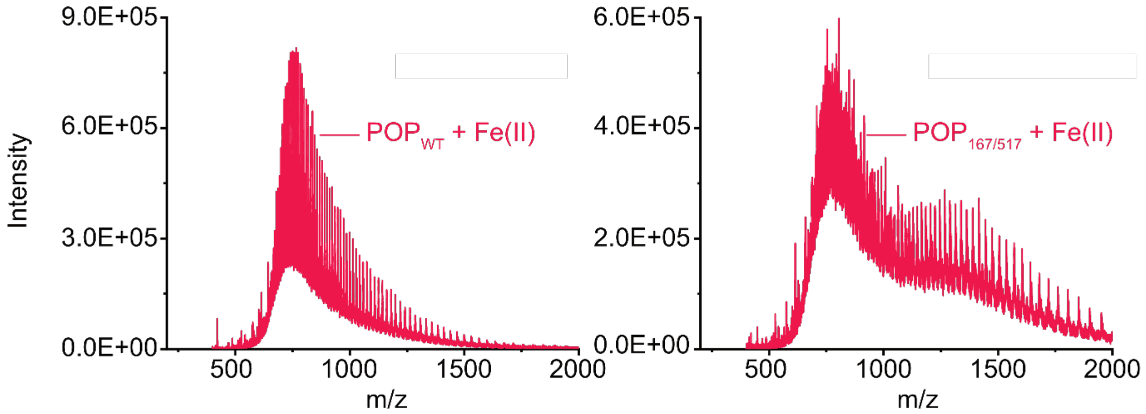

Supplementary Figure 12. Raw MS of POP variants treated Fe(II).
Raw MS data for POP_{WT} and $POP_{167/517}$ after treatment of the apo proteins with 5 equivalents of $(NH_4)_2Fe(SO_4)_2 \cdot 6H_2O$ and subsequent removal of excess Fe(II).

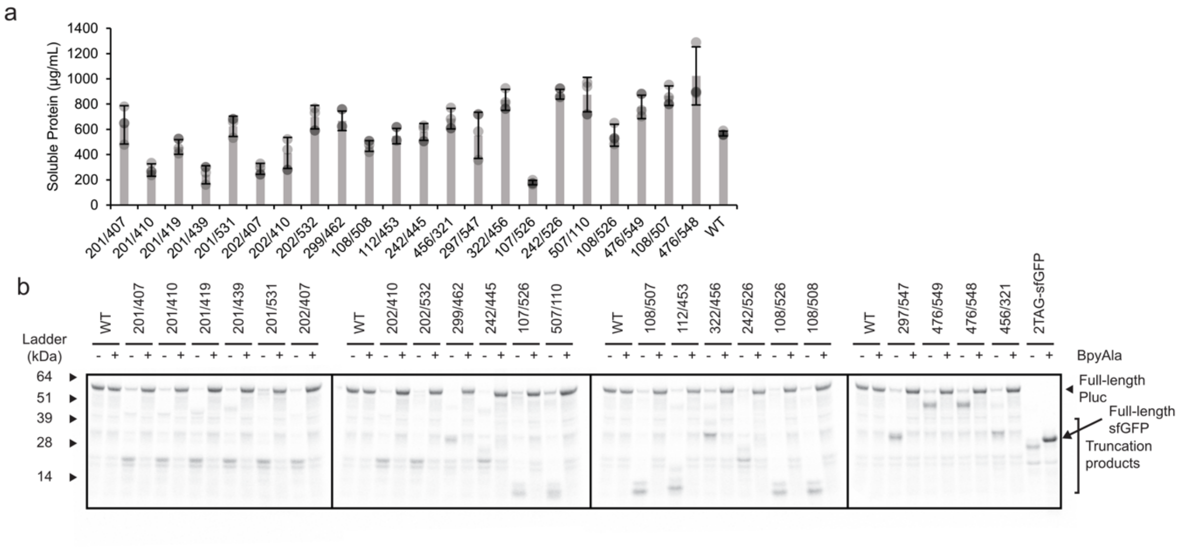

Supplementary Figure 13. BpyAla incorporation into Pluc variants.

BpyAla pairs can be incorporated into Pluc variants **(a)** Soluble expression of all Pluc variants shows yields comparable to WT Pluc in most variants. Data represent mean ± standard deviation from n=3 replicates, and results are representative of two independent experiments. **(b)** Autoradiograms of ^{14}C-labelled Pluc variants synthesized in CFPS show that the major product of reactions including BpyAla is at similar sizes as WT Pluc. Only a minor amount of full-length product is made in the absence of BpyAla.

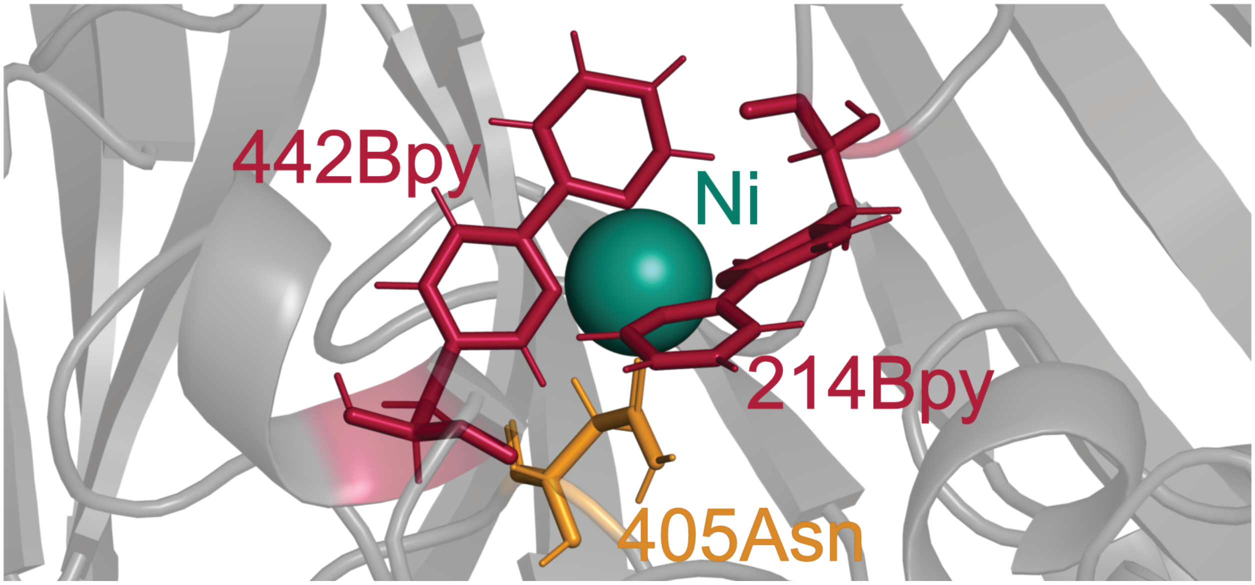

Supplementary Figure 14. MD simulation of $POP_{214/442}$.
MD simulations of variant $POP_{214/442}$ shows coordination of the Ni(II) ion to native asparagine (N405) residue.

Synthetic Procedures

General Materials and Methods

Unless otherwise noted, all reagents were obtained from commercial suppliers and used without further purification. Deuterated solvents were obtained from Cambridge Isotope Laboratories, Inc (Tewksbury, MA). Silicycle silica gel plates (250 mm, 60 F254) were used for analytical TLC, and preparative chromatography was performed using SiliCycle (Quebec City, QC) SiliaFlash silica gel (230-400 mesh).

Column chromatography was carried out using Silicycle 230-400 mesh silica gel. ^{1}H was recorded at 400 MHz on a Varian 400 MHz Inova NMR Spectrometer and chemical shifts are reported relative to residual solvent peaks. Chemical shifts are reported in ppm and coupling constants are reported in Hz.

Synthesis of BpyAla

R,S-(2,2'-Bipyridin-5-yl)alanine was synthesized using protocol modified from reported methods.[4]

1-(2-pyridylacetyl)pyridinium iodide

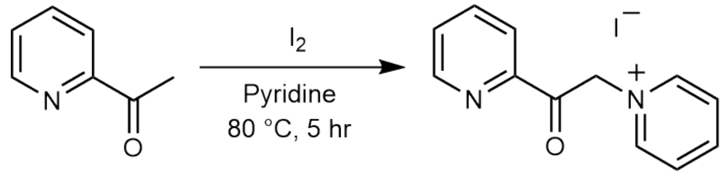

I_2 (10 g, 40 mmol) was dissolved in 60 mL pyridine and heated to 70 °C for 30 min. 2-acetylpyridine (4.84 g, 40 mmol) was added and the solution was stirred at 80 °C for 5 hr. After the mixture cooled, the solution was filtered off and washed with ethanol several times. The greenish solid was dried in vacuo overnight to afford the target compound (8.17 g, 62%). The product was used for the next step of the synthesis without any further purification.

5-methyl-2,2'-bipyridine

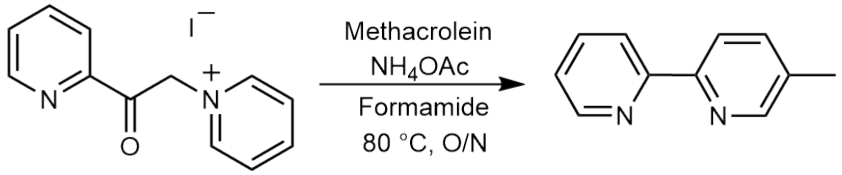

Methacrolein (1.89 g, 27.05 mmol) and NH_4OAc (5.79 g, 75 mmol) were sequentially added to a solution of 1-(2-pyridylacetyl)pyridinium iodide (8.17 g, 25 mmol) in formamide (80 mL). The mixture was stirred at 80 °C overnight. Then, the crude mixture was cooled and extracted with DCM (3 x 50 mL). The combined organic layers were washed with brine (200 mL), dried over $MgSO_4$, filtered, and concentrated in vacuo. The crude product was purified by silica gel column chromatography (5% MeOH in CH_2Cl_2) to yield the product as a yellow oil (1.65 g, 39%). The collected spectra matched literature reports.[5] ^1H NMR (400 MHz, $CDCl_3$): δ 8.70 – 8.65 (m, 1H), 8.52 (d, J = 2.3 Hz, 1H), 8.39 (d, J = 8.2 Hz, 1H), 8.31 (d, J = 8.3 Hz, 1H), 7.82 (td, J = 7.8, 1.9 Hz, 1H), 7.69 – 7.61 (m, 1H), 7.30 (ddd, J = 7.5, 4.8, 1.3 Hz, 1H), 2.41 (s, 3H).

^1H NMR spectrum (400 MHz, $CDCl_3$):

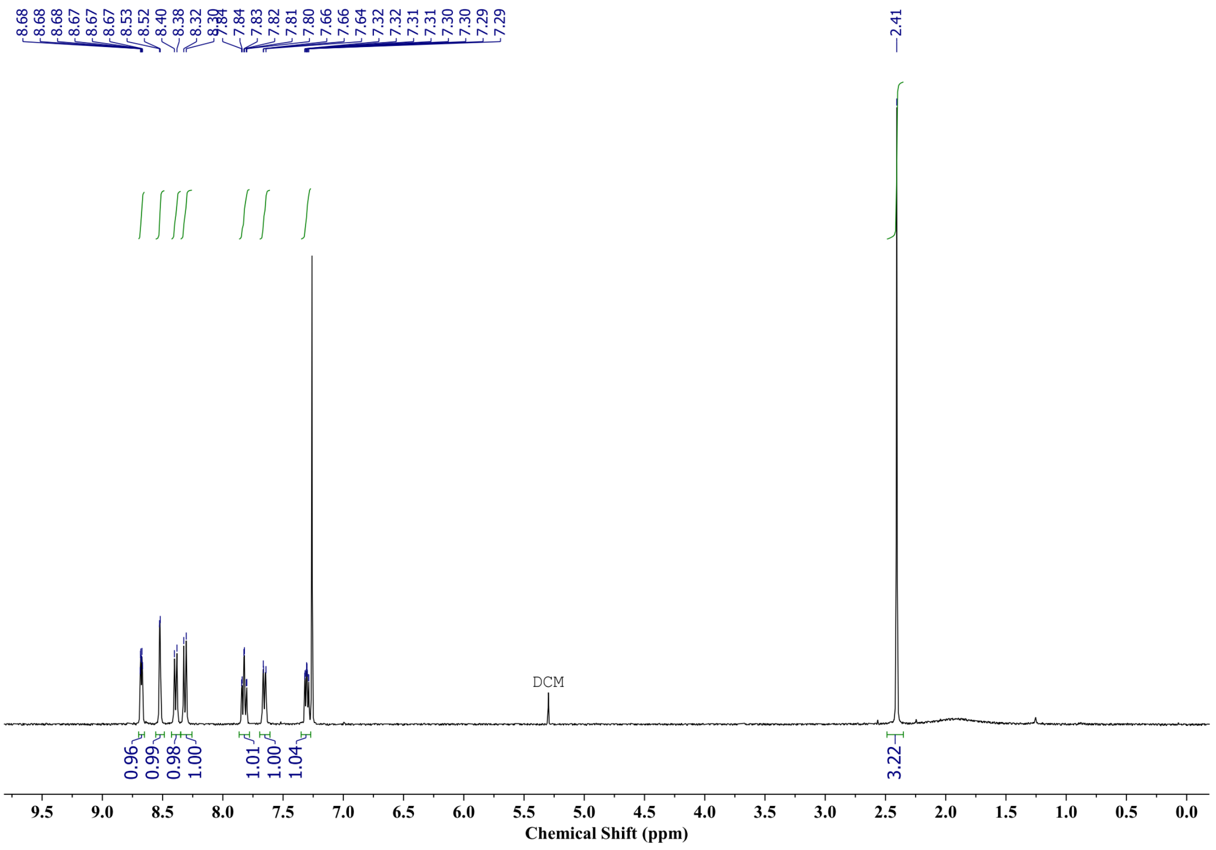

5-(bromomethyl)-2,2'-bipyridine

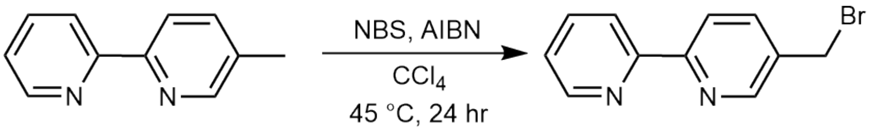

5-methyl-2,2'-bipyridine (1.65 g, 9.7 mmol), NBS (1.73 g, 9.7 mmol), and AIBN (400 mg, 2.43 mmol) were heated to 45 °C for 24 h in dry CCl_4 (80 mL). Then, the suspension was filtered. After removal of the solvent in vacuo, n-hexane (40 mL) was added immediately to the remaining oil. The mixture was stirred for 1 hr, wherein a white solid precipitated. The crude product was filtered off, washed with n-hexane (40 mL), yielding a white solid as the product (1.67 g, 69%). The collected spectra were consistent with a mixture of un-, mono-, and di-brominated product, which was used without further purification.[6] ^1H NMR (400 MHz, $CDCl_3$): δ 8.69 (d, J = 2.8 Hz, 2H), 8.48 – 8.26 (m, 2H), 7.84 (ddd, J = 15.6, 8.1, 2.1 Hz, 2H), 7.33 (ddd, J = 7.6, 4.7, 1.2 Hz, 1H), 4.54 (s, 2H).

^1H NMR spectrum (400 MHz, $CDCl_3$):

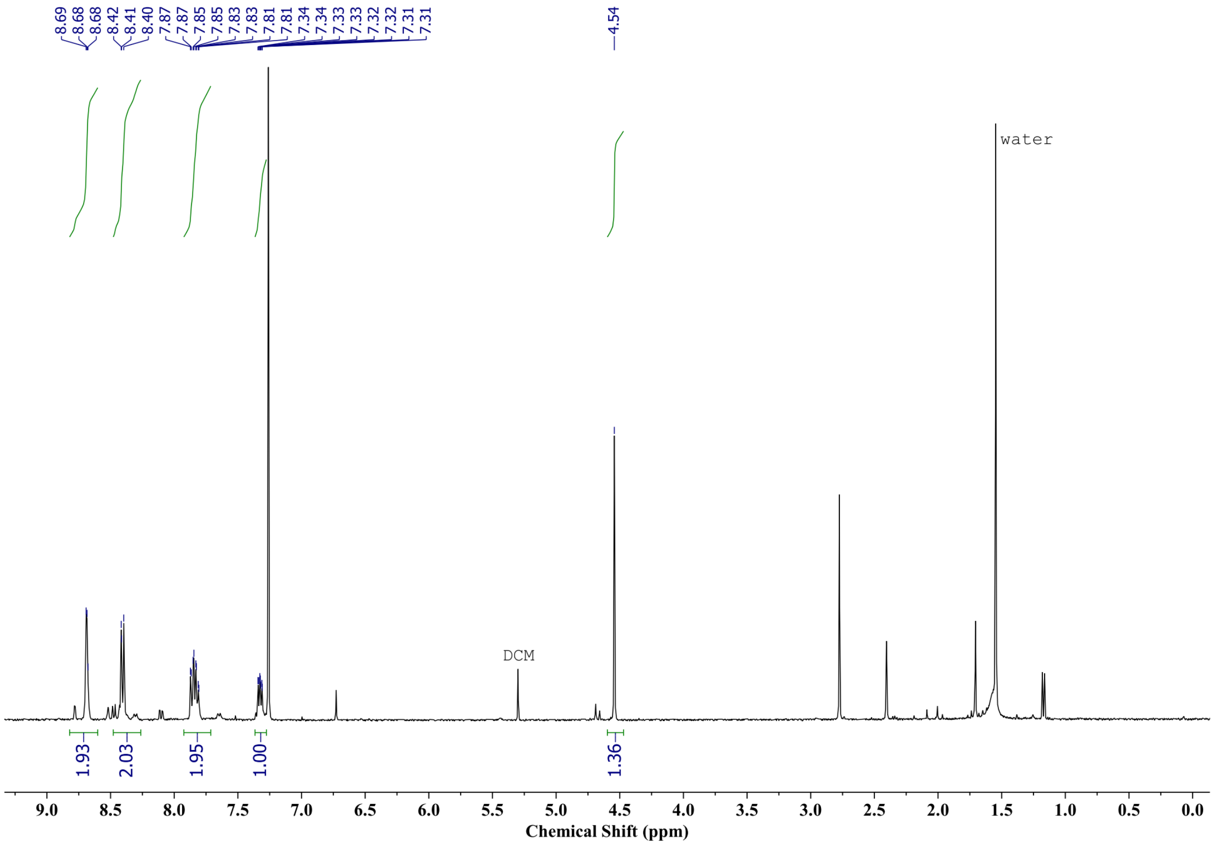

Diethyl-2-(2,2'-bipyridin-5-ylmethyl)-2-acetamidomalonate

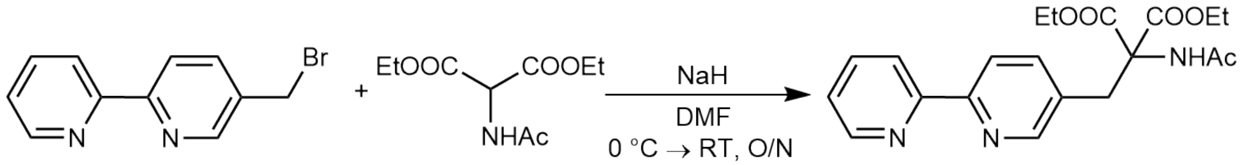

To a solution of diethyl acetamidomalonate (1.606 g, 7.4 mmol) and NaH (177 mg, 7.4 mmol) in anhydrous DMF (50 mL), 5-(bromomethyl)-2,2'-bipyridine (1.67 g, 6.73 mmol) was added at 0 °C under an N_2 atmosphere. The reaction mixture was stirred at room temperature overnight. The solvent was evaporated in vacuo and the residue was purified by silica gel column chromatography (CH_2Cl_2/MeOH 99:2) to give the product as a white solid[7] (1.06 g, 41%). ^{1}H NMR (400 MHz, $CDCl_3$): δ 8.65 (ddd, J = 4.9, 1.9, 1.0 Hz, 1H), 8.35 – 8.25 (m, 3H), 7.79 (td, J = 7.7, 1.8 Hz, 1H), 7.46 (dd, J = 8.2, 2.3 Hz, 1H), 7.29 (ddd, J = 7.5, 4.8, 1.3 Hz, 1H), 6.62 (s, 1H), 4.27 (qd, J = 7.1, 2.4 Hz, 4H), 3.71 (s, 2H), 2.05 (s, 4H), 1.29 (t, J = 7.1 Hz, 7H).

^{1}H NMR spectrum (400 MHz, $CDCl_3$):

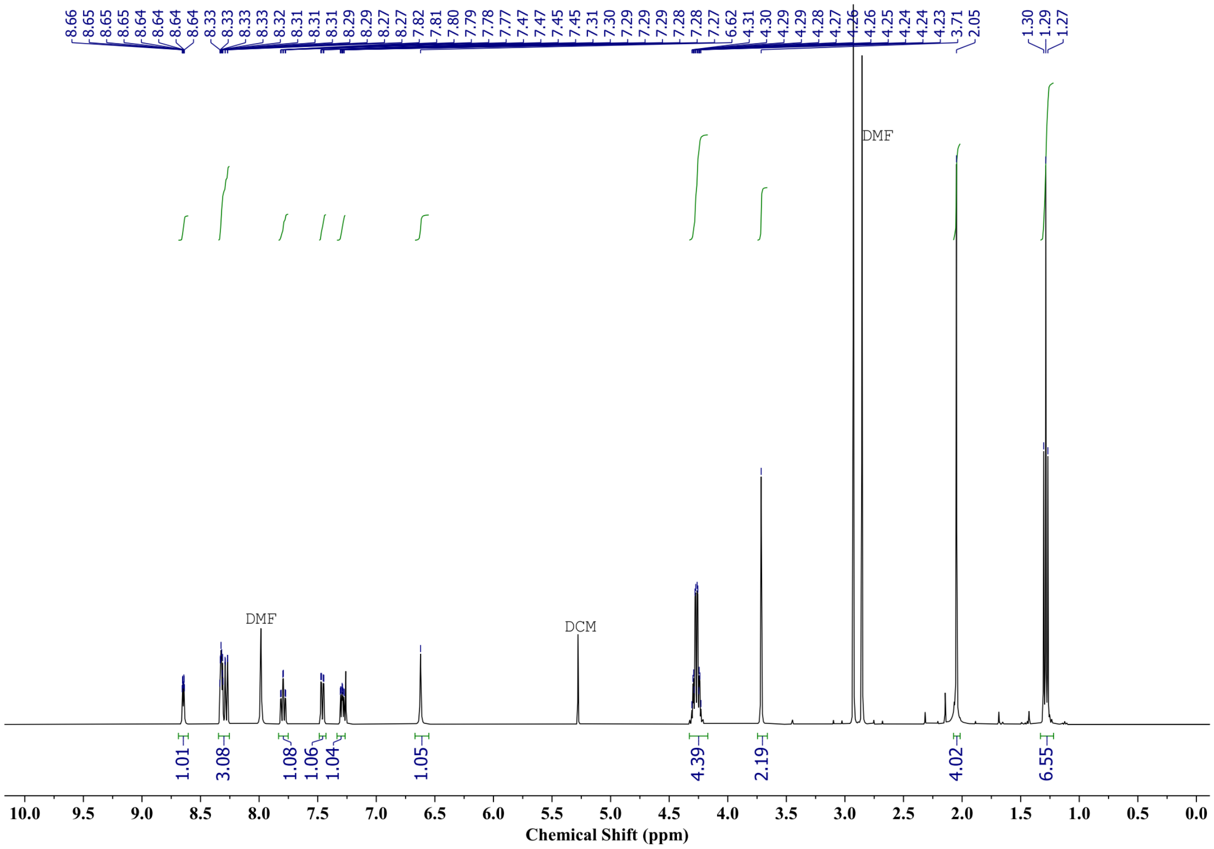

R,S-(2,2'-Bipyridin-5-yl)alanine

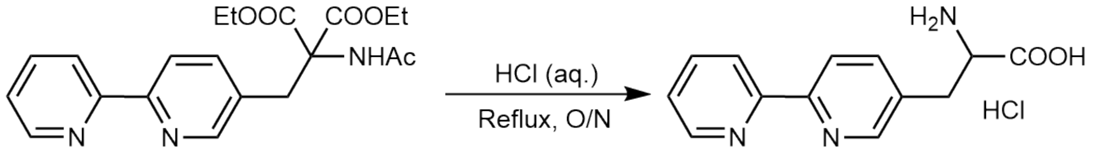

A suspension of diethyl-2-(2,2’-bipyridin-5-ylmethyl)-2-acetamidomalonate (1.06 g, 2.75 mmol) in aqueous HCl (60 mL, 37% in water) was heated to reflux overnight. The solvent was evaporated in vacuo to afford R,S-(2,2'-Bipyridin-5-yl)alanine as a pale yellow salt[8] (768 mg, 99%). ^{1}H NMR (400 MHz, DMSO-d$_6$): δ 8.70 (d, J = 4.6 Hz, 2H), 8.55 (d, J = 8.1 Hz, 1H), 8.47 – 8.39 (m, 1H), 8.35 (dd, J = 8.1, 2.9 Hz, 1H), 8.08 (d, J = 7.8 Hz, 1H), 7.88 – 7.80 (m, 1H), 4.32 – 4.23 (m, 1H), 3.40 – 3.17 (m, 2H).

^{1}H NMR spectrum (400 MHz, DMSO-d$_6$):

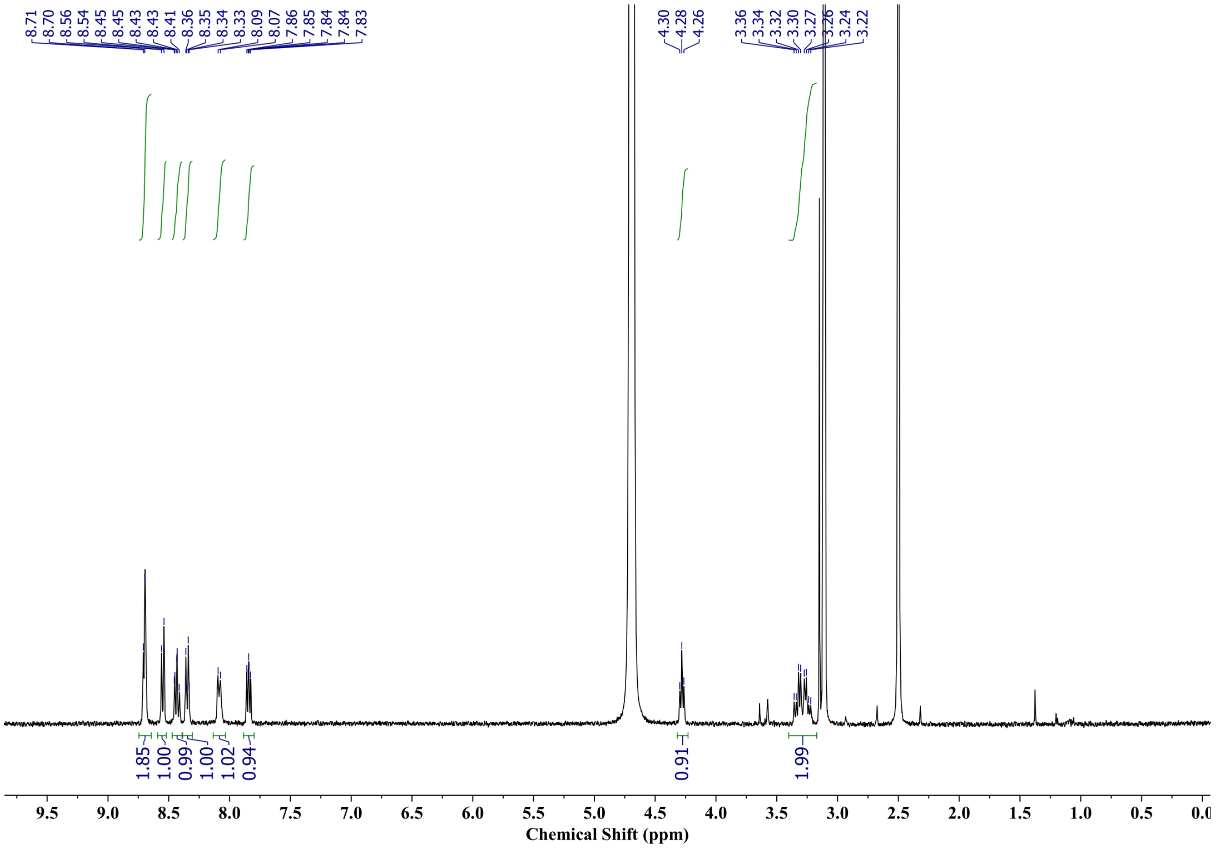

Computational Procedures

Force Field Parameterization

To capture the binding ability of Bpy in the present of metal ions (Ni^{2+}, Zn^{2+}, etc.), we conducted potential of mean force (PMF)[9] calculations from molecular dynamics simulations of Bpy molecules and metal ions in water solvents. Furthermore, we calculated the binding constant from the one-dimensional PMF. From the experimental value of the binding constant, we parameterized the non-bonded fix parameters for the CHARMM force field[10]. Experimentally, the equilibrium association constant of metal ions (Ni^{2+}, Zn^{2+}, etc.) and Bpy is available from the literature.[11]

For example, Supplementary Table 3 shows the K_{eq} of the reaction of Zn^{2+} + Bpy <---> $Zn(Bpy)^{2+}$ and Zn^{2+} + 2 Bpy <---> $Zn(Bpy)_2{}^{2+}$ are approximately 5.34 and 9.96 at 25 °C and 1.0 atm.

If Zn^{2+} + Bpy <---> $Zn(Bpy)^{2+}$, $K_{eq}^{2B} = \frac{[Zn(Bpy)^{2+}]}{[Zn^{2+}][Bpy]}$, where $\log\left(K_{eq}^{2B}\right) = 5.34$

Zn^{2+} + 2 Bpy <---> $Zn(Bpy)_2{}^{2+}$, $K_{eq}^{3B} = \frac{[Zn(Bpy)_2^{2+}]}{[Zn^{2+}][Bpy]^2} = \frac{[Zn(Bpy)^{2+}]}{[Zn^{2+}][Bpy]} \times \frac{[Zn(Bpy)_2^{2+}]}{[Zn(Bpy)^{2+}][Bpy]} = K_{eq}^{2B} \cdot K_{eq}^{*}$, where $\log\left(K_{eq}^{3B}\right) = 9.96$. Then,

$Zn(Bpy)^{2+}$ + Bpy <---> $Zn(Bpy)_2{}^{2+}$, $K_{eq}^{*} = \frac{[Zn(Bpy)_2^{2+}]}{[Zn(Bpy)^{2+}][Bpy]} = \frac{K_{eq}^{3B}}{K_{eq}^{2B}}$, where $\log\left(K_{eq}^{*}\right) = \log\left(K_{eq}^{3B}\right) - \log\left(K_{eq}^{2B}\right) = 9.96 - 5.34 = 4.62$

We transferred the association constants into the form of 2-Body binding:

$K_{eq} = \frac{1}{1661}\int_0^{R_c} 4\pi r^2 e^{-\beta W(r)} dr$,[12] where 1661 is the pre-factor of unit conversion, $\beta = \frac{1}{k_B T} = \frac{1}{0.593 Kcal/mol}$ and $W(r)$ is the one-dimensional potential of mean force (PMF).

The task is to calibrate how different values of non-bonded interactions (NBFiX) affect the above-mentioned binding constants and to guide selection of the suitable one for $M^{II}(Bpy)_n$ molecules.

We generate several 50 x 50 x 50 $Å^3$ water boxes which contain 3901 water molecules and 1 or 2 Bpy molecules for each M^{2+} and 2 Cl^- (to keep the electronically neutralization of the system), individually. Wherein, the nonbonded interaction between cations and water was adopted as in the literature.[13] We used replicas exchange MD for PMF on 24 windows, each window is distance away 0.4 Å for M^{2+} and the Bpy molecules running for 2 ns. In the case where M=Zn, for the purpose of verifying the duration of simulation is long enough for statistically sampling, we ran the best set of parameters for 4 ns, 6 ns, and 8 ns, separately, for each window (Supplementary Figure 15).

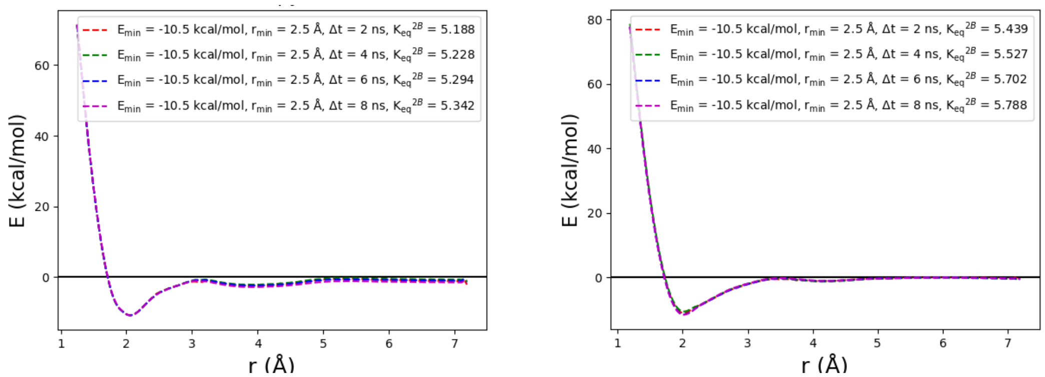

Supplementary Figure 15. PMF calculations with different simulation times.
One dimensional PMF for Zn^{2+} and Bpy molecules in water under the same combinations of ε_{min} (-10.5 kcal/mol) and r_{min} (2.5 Å). Simulation time was varied from 2 to 8 ns.

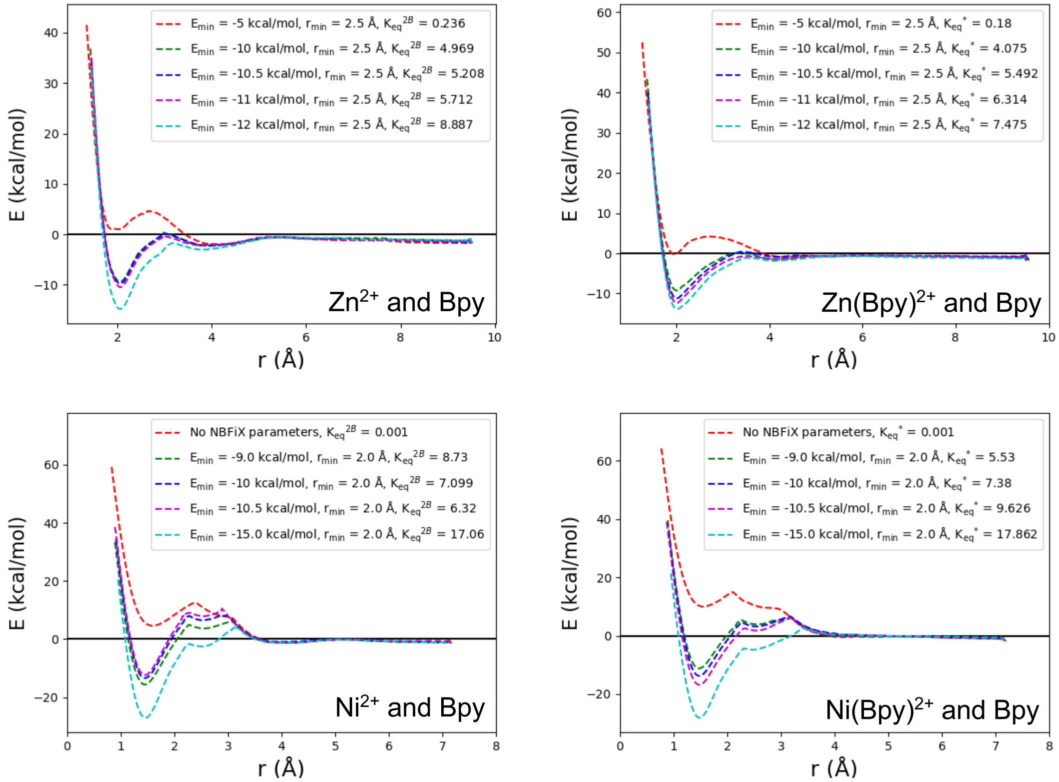

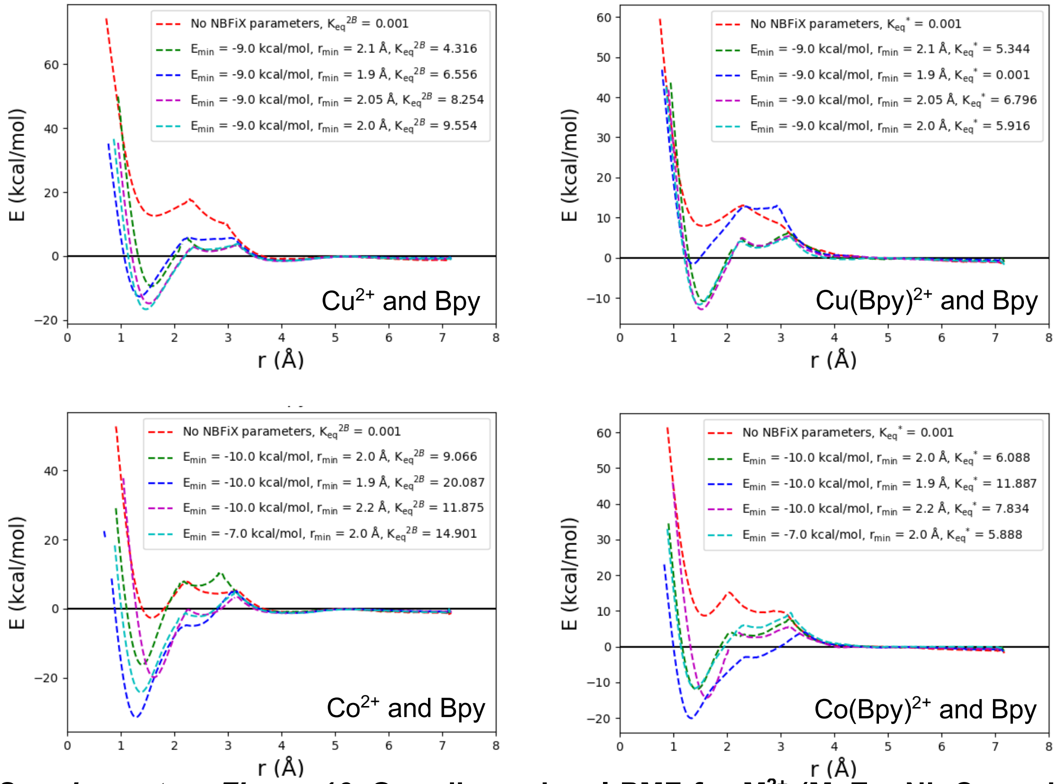

***Supplementary Figure 16.* One dimensional PMF for M^{2+} (M=Zn, Ni, Cu and Co) and Bpy molecules in water under different combinations of ε_{min} and r_{min}.** The best combinations of ε_{min} and r_{min} to describe both K_{eq}^{2B} and K_{eq}^{*} in 2 ns simulations were selected and the resulting NBFiX parameters are summarized in Supplementary Table 3. It is apparent that the different ε_{min} and r_{min} values' effects on the binding constants is a combinatory result.

Supplementary Table 3. Summary of NBFiX Parameters and equilibrium constants for M^{2+} and Bpy.

	Ni^{2+}		Zn^{2+}		Cu^{2+}		Co^{2+}	
	[a]Exp.	[b]Comp.	[a]Exp.	[c]Comp.	[a]Exp.	[d]Comp.	[a]Exp.	[e]Comp.
$\log(K_{eq}^{2B})$	7.06	7.099	5.34	5.301	8.52	8.254	5.81	9.066
$\log(K_{eq}^{*})$	6.94	7.38	4.62	5.887	5.78	6.796	5.49	6.088

[a]Experimental values were taken from the literature.[11] [b]Computational thermodynamic values for Ni(II) were calculated using r_{min} = 2.0 Å and ε_{min} = -10.0 kcal/mol. Parameters were taken from 2 ns PMF simulations. [c]Computational thermodynamic values for Zn(II) were calculated using r_{min} = 2.5 Å and ε_{min} = -10.5 kcal/mol. Parameters were taken from 8 ns PMF simulations. [d]Computational thermodynamic values for Cu(II) were calculated using r_{min} = 2.05 Å and ε_{min} = -9.0 kcal/mol. Parameters were taken from 2 ns PMF simulations. [e]Computational thermodynamic values for Co(II) were calculated using r_{min} = 2.0 Å and ε_{min} = -10.0 kcal/mol. Parameters were taken from 2 ns PMF simulations.

MD Simulations of POP
MD simulations were performed for the wildtype (WT) and 167/517 BpyAla ($POP_{167/517}$) mutated *Pfu* POP enzymes. Initial configurations for the WT simulations were taken from the corresponding crystal structure (PDB: 5t88). For the WT, chain B with a lower B-factor in the crystal structures was used to generate the simulation system. For the BpyAla mutations, their initial structures were the WT fused using VMD[14]. The systems were bathed in 0.15M KCl solution using the *Solution Builder* Module in CHARMM-GUI[15]. The systems were roughly 100 ×100 ×100 $Å^3$ in dimension and contained ~110,000 atoms. The periodic boundary conditions were counted using the particle-mesh Ewald method with an automatic generated grid size.

Once the simulation systems were generated, they were subjected to equilibrating at 358.15 K. The system was first equilibrated in an NVT ensemble for 10 ns. The equilibration simulations were performed using NAMD2.14 GPU acceleration version package[16] on Nvidia's P100 GPUs. After equilibration, the systems were simulated for 1000 ns each in an NPT ensemble with temperature set to 358.15 K and the isotropic pressure set to 1 atm. The Langevin thermostats with a damping coefficient of 1 ps^{-1} are used to keep the temperature constant. The cutoff of the van der Waals interactions and short-range electrostatic interactions were set to ~12 Å as suggested by the guesser script. The additive C36 force field was used in all the simulations performed here.[17–20] The force field parameters for the BpyAla itself was from CGENFF[21], and the interaction of Bpy and cations were described by the parameterized NBFiX from above in *Force Field Parameterization* (*vide supra)*.

MD simulations were performed for the different BpyAla mutated *Pfu* POP enzymes. The initial structure of the different BpyAla variant was constructed from the crystal structure of POP, then mutate the according amino acids into BpyAla molecules using VMD[14]. For example, the variant $POP_{214/442}$ was constructed by mutating TRP_{214} and HSD_{442} into BpyAla. A metal divalent cation was placed at the proximity of the mutated ncAA as seen in Supplementary Figure 14. All the structures were bathed in 0.15M KCl solution using the *Solution Builder* Module in CHARMM-GUI[15]. The systems were roughly 100 ×100 ×100 $Å^3$ in dimension and contained ~110,000 atoms. The periodic boundary conditions were counted using the particle-mesh Ewald method with an automatic generated grid size.

Once the simulation systems were generated, they were subjected to equilibration at 300 K. The system was first equilibrated in an NVT ensemble for 10 ns. The equilibration simulations were performed using NAMD2.14 GPU acceleration version package[16] on Nvidia's P100 GPUs. After equilibration, the systems were simulated for 100 ns each in an NPT ensemble with temperature set to 300 K and the isotropic pressure set to 1 atm. The Langevin thermostats with a damping coefficient of 1 ps^{-1} are used to keep the temperature constant. The cutoff of the van der Waals interactions and short-range electrostatic interactions were set to ~12 Å as suggested by the guesser script. The additive C36 force field was used in all the simulations performed here.[17–20] The force field parameters for the BpyAla itself was from CGENFF[21], and the interaction of Bpy and cations were described by the parameterized NBFiX from above in *Force Field Parameterization* (*vide supra)*.

MD Simulations of Luciferase
We performed MD simulations on wildtype (WT) (round 1) (PDB: 4G36) and cross-linked (round 2) (PDB: 4G37) luciferase. Initial configuration for the WT simulation was taken from the corresponding crystal structure[22]. The system was bathed in 0.15 M KCl solution using the *Solution Builder* Module in CHARMM-GUI[15]. The systems were roughly 100 ×100 ×100 $Å^3$ in

dimension and contained ~110,000 atoms. The periodic boundary conditions were counted using the particle-mesh Ewald method with an automatic generated grid size. Once the simulation system was generated, it was subjected to equilibration at 300 K. The system was first equilibrated in an NVT ensemble for 10 ns. The equilibration simulation was performed using NAMD2.14 GPU acceleration version package[16] on Nvidia's P100 GPUs. After equilibration, the system was simulated for 100 ns in an NPT ensemble with temperature set to 300 K and the isotropic pressure set to 1 atm. The Langevin thermostats with a damping coefficient of 1 ps^{-1} are used to keep the temperature constant. The cutoff of the van der Waals interactions and short-range electrostatic interactions were set to ~12 Å as suggested by the guesser script. The additive C36 force field was used in all the simulations performed here.[17–20]

Due to only running the WT simulation for 100 ns, not very significant conformational changes were found through the WT simulation. We formed another initial cross-linked configuration based on the backbone structure of the cross-linked one, while mutating the all the residues to the WT one[22]. The system was bathed in 0.15 M KCl solution using the *Solution Builder* Module in CHARMM-GUI[15]. The systems were roughly 100 ×100 ×100 $Å^3$ in dimension and contained ~110,000 atoms. The periodic boundary conditions were counted using the particle-mesh Ewald method with an automatic generated grid size. Once the simulation system was generated, it was subjected to equilibration at 300 K. The system was first equilibrated in an NVT ensemble for 10 ns. The equilibration simulation was performed using NAMD2.14 GPU acceleration version package[16] on Nvidia's P100 GPUs. After equilibration, the system was simulated for 100 ns in an NPT ensemble with temperature set to 300 K and the isotropic pressure set to 1 atm. The Langevin thermostats with a damping coefficient of 1 ps^{-1} are used to keep the temperature constant. The cutoff of the van der Waals interactions and short-range electrostatic interactions were set to ~12 Å as suggested by the guesser script. The additive C36 force field was used in all the simulations performed here.[17–20]

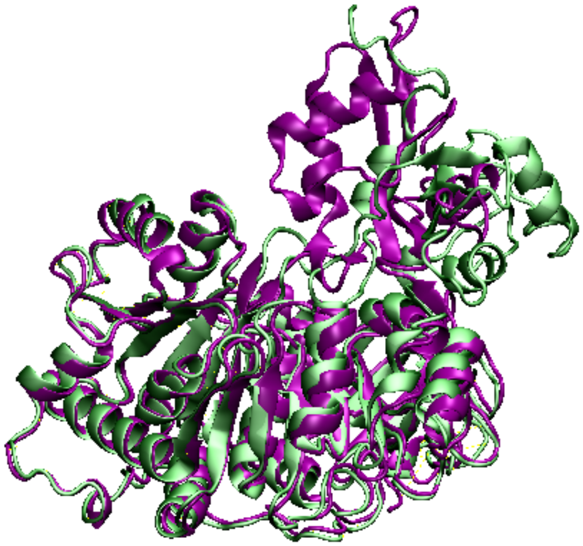

Supplementary Figure 17. MD-simulations luciferase structures.
MD-simulated structures of luciferase generated with starting coordinates obtained from reported crystal structures of WT (green) and cross-linked (purple) variants.

BpyAla Site Selection in POP

We previously reported that WT POP undergoes large-scale domain opening and closing in MD simulations[23]. We further selected the proper site of ncAAs mutation according to the opening and closing structures of POP from that MD simulation. Firstly, by taking the Bpy structure and cations into the quantum mechanics (QM) simulation, we obtained the distance value (~10.5 Å) between those BpyAla linkages (Supplementary Figure 18). According to the QM simulation, the ideal (equilibrated) distance for this complex acting as a linker would be around 10.5 Å (this might slightly be change in MD simulation).

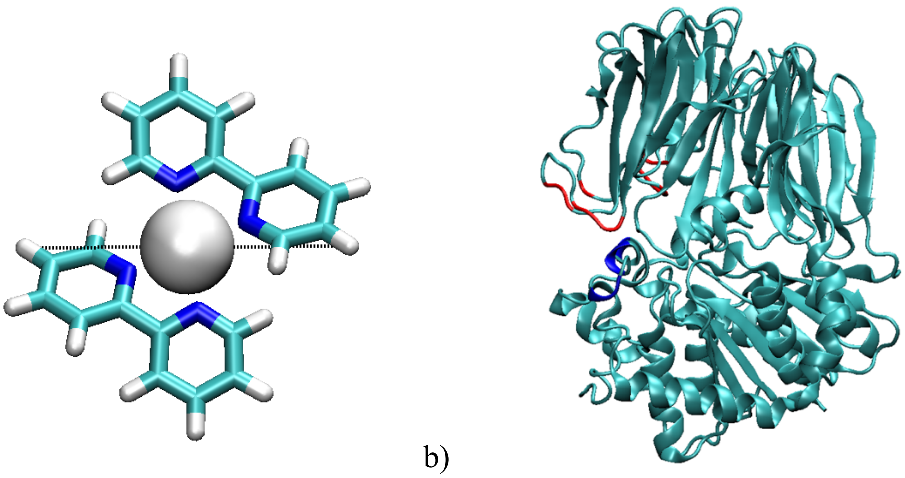

a) b)

Supplementary Figure 18. Selection of sites for BpyAla incorporation in POP.
a) Distance between the BpyAla linkage indicated from QM simulation. **b)** Red and blue sites indicate where BpyAla pairs were introduced on POP.

We applied two selection criterions on the closing structure of POP from MD simulation: 1) The distance should be 10 Å < dij_c ± std_c < 11 Å, where dij_c stands for the distance of C-β atoms between *i*-th residue and *j*-th residue from the closed POP conformation in MD simulations, std_c is the root mean square displacement (fluctuations) of the distance averaged over ±5 ns around the frame of the closing confirmation. 2) residues are water exposed. Through analysis of MD simulation, the identified residues are shown in Supplementary Table 1.

BpyAla Site Selection in Luciferase

Since we performed two MD simulations for luciferase, we conducted two rounds of BpyAla site selection. Round 1 was based on 100 ns MD simulation of the WT variant. We used the same selection criterion as for POP: 1) The distance should be 10 Å < dij_c ± std_c < 11 Å, where dij_c stands for the distance of C-β atoms between *i*-th residue and *j*-th residue from the closed WT luciferase conformation in MD simulations, std_c is the root mean square displacement (fluctuations) of the distance averaged over ±5 ns around the frame of the closing conformation. 2) residues are water exposed. We also excluded certain sites (i.e., Ser198, Gly200, Lys206, His245, Gly316, Tyr340, Glu344, Glu389, Tyr401, Ser420, Gly421, Asp422, Arg437, Lys443, Gly446, Gln448, Glu455, Glu479, and Lys529) since they are highly conserved, and mutation could result in diminished activity. Round 2 was based on 100 ns MD simulations of the mutated cross-link. Then the rest of criteria are the same as Round 1: 1) The distance should be 10 Å < dij_c ± std_c < 11 Å, where dij_c stands for the distance of C-β atoms between *i*-th residue and *j*-th residue from the closed cross-linked luciferase conformation in MD simulations, std_c is the root mean square displacement (fluctuations) of the distance averaged over ±5 ns around the frame of the closing conformation. 2) residues are water exposed. We also excluded certain sites (*vide supra*) since they are highly conserved, and mutation could result in diminished activity. Through analysis of MD simulations, the identified residues are shown in Supplementary Table 4.

Supplementary Table 4. Residues chosen for mutation to BpyAla in luciferase.

**Residue #**	**Residue *i***	**Residue #**	**Residue *j***
Round 1			
201	SER	407	ALA
201	SER	410	ALA
201	SER	419	HSD
201	SER	439	LYS
201	SER	531	ASP
202	THR	407	ALA
202	THR	410	ALA
202	THR	532	ALA
299	THR	462	PRO
Round 2			
108	ILE	508	THR
242	PRO	445	LYS
297	LYS	547	LYS
107	ASP	526	LEU
507	THR	110	ASN
476	ASP	549	LYS
108	ILE	507	THR
476	ASP	548	SER
112	ARG	453	GLU
456	SER	321	LYS
322	GLU	456	SER
242	PRO	526	LEU
108	ILE	526	LEU

High-Throughput Procedures and Screening

General Materials and Methods

All materials were purchased from Sigma unless otherwise noted. Aqueous media was prepared using Milli-Q water and sterilized either by autoclaving or by filtration. Antibiotics were prepared as 1,000x stocks and stored at -20 °C. Final concentrations for antibiotics used were: Kanamycin (Kan) at 50 µg/mL, Carbenicillin (Cb, IBI Scientific) at 100 µg/mL, and Chloramphenicol (Cm) at 34 µg/mL. *E. coli* cultures were handled using sterile technique under open flame. All primers were ordered from IDT. All PCR reactions were done with Q5 High-Fidelity Polymerase (NEB) using manufacturer's recommended protocols. DNA was quantified by absorbance at 260 nm using a Nanodrop 2000c (Thermo Scientific). All gels were imaged in a Bio-Rad Gel Doc XR+.

DNA Cloning and Preparation Method

All cloning was performed in *E. coli* DH10B cells (ThermoFisher). pJL1 was used as the vector backbone for all CFPS expression plasmids. All plasmids used for CFPS were purified using ZymoPURE II Midiprep kits.

All POP variants were initially ordered with C-terminal 10X His tags from Twist Biosciences, which were exchanged for the StrepII tag by Gibson Assembly. Briefly, a ~250 bp gBlock containing the StrepII-tag flanked by POP and by the PJL1 backbone was ordered from IDT. The PJL1 backbone with ~25 bp of homology to the gBlock was amplified using Q5 High-Fidelity DNA Polymerase (NEB) according to manufacturer's protocol. Melting temperatures for primers were calculated using NEB Tm Calculator (https://tmcalculator.neb.com/#!/main). 2.5 µL of PCR products were mixed with 6X loading dye (NEB), loaded onto a 1% w/v agarose gel with SYBR Safe (Apex Bio) that was run at 110V for 45 minutes, and imaged to verify size. A homemade 3X Gibson Assembly mixture (75 mM Tris-HCl pH 7.5, 7.5 mM $MgCl_2$, 0.15 mM dNTPs, 7.5 mM DTT, 0.75 mM NAD, 0.004 U/µL T5 Exonuclease (NEB), 0.025 U/µL Phusion Polymerase (NEB), 4 U/µL Taq DNA Ligase (NEB), 3.125 µg/mL ET SSB (NEB)) was mixed with 25 ng of linearized backbone with a 3X molar excess of the gBlock and incubated for 1 h at 50 °C to assemble the plasmid. The product was transformed into *E. coli* and grown overnight on a LB-Kan agar (BD) plate at 37 °C. Single colonies were inoculated into 5 mL of LB-Kan and grown overnight at 37 °C with 250 RPM shaking. DNA was purified using the ZymoPURE II Miniprep kit following manufacturer's instructions and sequence-confirmed by Sanger Sequencing (GeneWiz). Sequence confirmed constructs were re-transformed and midiprepped following manufacturer's instructions.

pET-BpyRS was similarly assembled by using Gibson Assembly to ligate an IDT gBlock containing the BpyRS gene to pETBCS.NS, a pET variant with redesigned restriction enzyme sites.[24] Cb was used rather than Kan for this cloning scheme.

BpyRS purification

pET-BpyRS plasmid was transformed into chemically competent BL21(DE3) Star (ThermoFisher) following the manufacturer's protocols. Cells were plated onto LB-Cb and incubated overnight at 37 °C. A 20 mL overnight culture of LB-Cb was inoculated with a single colony at 37 °C with 250 RPM shaking the next day. 1 L of Overnight Express TB Medium (Millipore) was prepared by following the manufacturer's instructions and supplemented with Cb. The next day, the Overnight Express TB Medium was inoculated with 5 mL of the overnight culture and grown at 37 °C for ~ 16 hours.

Cells were harvested by centrifugation (Beckman Coulter Avanti J-26) at 5,000 x g for 15 min at 4 °C. Pellets were washed in 25 mL Buffer 1 (300 mM NaCl, 50 mM NaH_2PO_4 pH 8.0) and re-pelleted by centrifugation. Cells were resuspended in Buffer 1 supplemented with 10 mM imidazole pH 8.0 and lysed by homogenization (Avestin B3) at ~20,000 PSI. Lysate was treated

with Benzonase (Millipore) at room temperature with shaking for 30 min. Insoluble parts were pelleted by centrifugation at 20,000 x g for 10 min at 4 °C. Clarified lysate was incubated with 10 mL of pre-equilibrated NiNTA resin (Qiagen) for 1 h at 4 °C. Resin was loaded onto a gravity-flow column and washed twice with 45 mL of Buffer 1 with 20 mM imidazole pH 8.0. BpyRS was eluted with 30 mL of Buffer 1 with 0.5 M imidazole pH 8.0 and collected in 1.5 mL fractions. Protein containing fractions, as determined by absorbance at 280 nm measured by Nanodrop, were combined into a Slide-a-Lyzer Dialysis Cassette (3500 MWCO, ThermoFisher) and dialyzed against Buffer 1 with 40% v/v glycerol overnight. Dialysis buffer was exchanged the next day and dialyzed for another 6 hrs. Proteins were quantified by Bradford Assay (Bio-Rad), flash frozen in liquid nitrogen, and stored at -80 °C. Working aliquots, once thawed, were stored at -20 °C.

Protein purification was analyzed by SDS-PAGE. Samples were prepared by mixing 1 µL of protein, 3.75 µL of 4X NuPAGE LDS Sample Buffer (Invitrogen), 1.5 µL of 1 M DTT, and water to 15 µL. Samples were heat-treated at 95 °C for 10 min. Samples were loaded onto a NuPAGE 4-12% Bis-Tris Gel (Invitrogen) and run at 180V in 1X MES Buffer (Invitrogen). The SDS-PAGE gel was stained with InstantBlue Coomassie Protein Stain (Abcam) for 1 hr, destained in water overnight, and imaged.

Cell extract preparation

Electrocompetent 759.T7 was transformed with pEVOL-BpyRS, which was generously provided by Prof. Peter Schultz. Cells were plated onto LB-Cm and grown at 34 °C overnight. A single colony was inoculated into 100 mL of LB-Cm and grown at 34 °C overnight with 250 RPM shaking. The next day, three 1 L portions of 2xYTPG (16 g/L tryptone, 10 g/L yeast extract, 5 g/L NaCl, 7 g/L KH_2PO_4, 3 g/L K_2HPO_4, Cm) in 2.5L Tunair shake flasks were inoculated at OD_{600} = 0.075. The culture was grown at 34 °C with 250 RPM shaking. At OD_{600} = 0.6, 1 mL of 1 M IPTG was added to induce T7 RNA Polymerase expression. At OD_{600} = 3.0, cells were harvested by pelleting at 5,000 x g for 14 min at 4 °C, washed three times by resuspending in cold S30 buffer (10 mM Tris-Acetate pH 8.2, 14 mM Mg Acetate, 60 mM K Acetate, 2 mM DTT) and pelleting at 10,000 x g for 1 min at 4 °C, flash frozen in liquid nitrogen, and stored at -80 °C.

Frozen cell pellets were thawed on ice and resuspended in 0.8 mL of S30 buffer per gram of wet cell mass. 1.4 mL of resuspended cells were transferred to a 1.5 mL microcentrifuge tube and were lysed by sonication (Q125, QSonica) using three 45 sec ON and 59 sec OFF cycles at 50% amplitude to a total of ~940 J, as reported previously.[25] 4 µL of 1M DTT was added directly after lysis. Insoluble components were pelleted by centrifugation at 12,000 x g for 10 min at 4C. Supernatant was collected and subjected to a run-off reaction at 37 °C for 1 h with 250 RPM shaking. Insoluble components appearing after run-off were pelleted by centrifugation at 10,000 x g for 10 min at 4 °C. The remaining supernatant was aliquoted into single-use portions, flash frozen in liquid nitrogen, and stored at -80 °C.

Cell-free protein synthesis (CFPS)

CFPS reactions were based on a modified PANOx-SP system that is described in previous publications.[26] Briefly, 15 µL reactions were carried out in 2 mL microcentrifuge tubes in conditions of 8-12 mM Magnesium Glutamate, 10 mM Ammonium Glutamate (MP Bio), 130 mM Potassium Glutamate, 1.2 mM ATP, 0.85 mM of GTP, CTP, and UTP each, 0.03 mg/mL Folinic Acid, 0.17 mg/mL tRNA (Roche), 0.4 mM NAD, 0.27 mM CoA, 4 mM Oxalic Acid, 1 mM Putrescine, 1.5 mM Spermidine, 57 mM HEPES pH 7.2, 33 mM Phosphoenolpyruvate (Roche), 13.33 ng/µL DNA with PJL1 backbone, 1 mM BpyAla (Toronto Research Chemicals cat. B399200), 0.5 mg/mL BpyRS, 30% v/v 759.T7 cell extract, and water to 15 µL. Each batch of 759.T7 extract was separately optimized to find the optimal Magnesium Glutamate concentration, which typically ranged between 8-12 mM. Blank CFPS reactions were made by replacing PJL1 DNA with water.

Reactions were incubated at 30 °C for 20 hours.

POP Purification for MS Analysis

CFPS reactions were scaled up to 75 µL in a 15 mL conical tube and performed as previously described. POP containing a C-terminal StrepII tag was purified from the CFPS reaction using Strep-Tactin XT Spin Columns (IBA Life Sciences) according to the manufacturer's protocol. The C-terminal StrepII tag prevents purification of truncation products. POP purity was assessed by SDS-PAGE. Proteins were quantified by Nanodrop using extinction coefficients and molecular weights calculated by ExPasy ProtParam (https://web.expasy.org/protparam/).

Supplementary Table 5. Theoretical masses for POP variants produced by CFPS.

Variant	Theoretical Mass (Da)
216/516	72387.91
159/513	72311.76
238/516	72416.91
215/520	72391.89
170/513	72368.86
158/514	72333.77
167/516	72401.93
191/520	72418.92
169/514	72392.84
214/442	72279.76
167/517	72389.88
158/512	72333.77
169/516	72418.92
237/439	72386.92
156/513	72292.76
161/516	72387.86
167/513	72325.83
169/512	72392.84
156/516	72368.86
238/442	72366.85
191/513	72352.86
159/512	72361.78
191/517	72416.91
215/524	72374.9
169/510	72368.86
159/517	72375.81
214/524	72302.79
WT POP	72152.6

Intact Protein Mass Spectrometry

Purified proteins were buffer exchanged into PBS (1.37 mM NaCl, 27 mM KCl, 100 mM Na_2HPO_4, 18 mM KH_2PO_4, pH 7.4) using Amicon Ultra-0.5 10 kDA MWCO Centrifugal filters. Purified proteins in PBS were then analyzed by LC-MS, as described in previous publications.[24] m/z data was deconvoluted between a mass range of 70,000 – 75,000 Da, as only full-length protein was expected to be purified.

Protein Quantification in CFPS by Scintillation Counting

Protein concentrations for POP and Pluc were quantified by measuring incorporation of ^{14}C leucine in TCA precipitable counts with a scintillation counter (MicroBeta^2, Perkin Elmer). 10 μM ^{14}C-leucine (Perkin Elmer) was added to CFPS reactions and proteins were synthesized as described previously. For POP, a crude purification prior to centrifugation was done by heat treating CFPS reactions at 75 °C for 15 minutes. Insoluble components of spent CFPS reactions were then pelleted by centrifugation at 20,000 x g for 10 minutes at 4 °C. 5 μL of the soluble supernatant was treated with 5 μL of 0.5 M KOH and incubated at 37 °C for 20 min. Samples were then spotted onto two fiberglass paper sheets (Filtermat A, Perkin Elmer) and dried under a heating lamp for at least 30 min. One filtermat was washed three times with 5% w/v TCA at 4 °C for 15 min, then in 100% Ethanol at room temperature for 15 min, and dried under heating lamp for at least 40 min. A melt-on solid scintillator (Meltilex A, Perkin Elmer) was applied onto both filtermats at 95 °C, and scintillation counts were measured. Soluble yields of protein were calculated as described previously.

Autoradiogram analysis

CFPS reactions were run as previously described in the "Protein Quantification in CFPS by Scintillation Counting" section. For luciferase expression tests, BpyAla was removed from the reaction mixture as necessary. 3 μL of the soluble portion of the CFPS reaction was analyzed by SDS-PAGE. Gels were dried between cellophane sheets with a vacuum gel drier (Hoefer GD2000) at 60 °C for 1 hr. Dried gels were applied to a photobleached storage phosphoscreen and exposed for three days. Autoradiograms were scanned using Typhoon FLA 7000 Imager.

High-Throughput Screening and Analysis of POP mutants

CFPS reactions were performed to synthesize all proteins, including WT POP, the entire panel of POP mutants, and 2TAG-sfGFP. Only StrepII-tagged enzymes were used for the high-throughput experiments. Blank reactions to dilute reactions to be equimolar were set up in parallel.

After reaction completion, CFPS reactions were heat-treated at 75 °C for 15 minutes as a crude purification. Insoluble components were pelleted at 20,000 x g for 10 minutes at 4 °C. The supernatant containing POP enzymes was diluted to 1 μM using blank CFPS reactions as diluent. 4.5 μL of POP enzymes were then mixed with 22.5 μL of 2X POP Buffer (60 mM HEPES pH 7.4, 1.6 M NaCl) 4.5 μL of 0 – 1 mM metal (Nickel(II) Sulfate Hexahydrate, Copper(II) Sulfate Pentahydrate, Cobalt(II) Chloride Hexahydrate, or Zinc(II) Chloride), and 13.5 μL of water. The mixture was equilibrated on ice for 2 hr. For reversibility screening, 1.8 μL of 50 mM EDTA pH 8.0 was added and then incubated for an additional hour on ice. During the incubations, 1.2 μL of 25 mM Z-Ala-Pro-pNA (BACHEM, cat. 4003575.0250) in DMSO was spotted into each well of a clear, flat-bottom 384-well plate (Grenier, cat. 781096) from a source plate (Beckman Coulter, cat. 001-14622) using the Echo 550. After incubation on ice, the reaction mixture was split into three 13.5 μL aliquots and pre-warmed to 30 °C. Using the Integra Viaflo, 10.8 μL of the reaction mixture was dispensed into the substrate-containing 384-well plate and mixed thoroughly. The plate was quickly spun down and read on a pre-warmed plate reader (BioTek Synergy H1 Microplate Reader) at 30 °C at 410 nm for two hours.

Kinetic curves were analyzed using the Python script provided below. Briefly, this script requires two input files: (i) A minimally formatted raw data CSV and (ii) a descriptor CSV that annotates reaction conditions for each well. The script calculates rate by conducting a linear regression over a sliding window of five timepoints and calculates an average and standard deviation between all replicates. Finally, it identifies and plots the maximum slope for each reaction condition.

```
"""
Created on Thu Apr 22 17:15:56 2021

@author: Kosuke
"""

#importing libraries for data analysis
import pandas as pd
import matplotlib.pyplot as plt
import numpy as np
from scipy.stats import linregress

###formatting for plotting
plt.rcParams['font.sans-serif']="Arial"
plt.rcParams['font.family']='sans-serif'
plt.rcParams['font.size'] = 7
plt.rcParams['axes.linewidth'] = 1
plt.rcParams['axes.spines.right'] = False
plt.rcParams['axes.spines.top'] = False
plt.rcParams['xtick.major.size'] = 1
plt.rcParams['ytick.major.size'] = 1
plt.rcParams['xtick.major.width'] = 1
plt.rcParams['ytick.major.width'] = 1
plt.rcParams["figure.figsize"] = [4.25,4.25]

#importing data and tidying it
rfudf = pd.read_csv('Fig3_Formatted_Raw_Data.csv')
##col_info is a csv that specifies the reaction in each well
##i.e. A1 - this enzyme, with this concentration of metal, this rep#
col_info = pd.read_csv('Fig3_Well_Info.csv')
#Arranging into tidydata format
melt = rfudf.melt(id_vars = 'Time',
                  var_name = 'Well',
                  value_name = 'rfu')
full_data = pd.merge(melt,
                col_info,
                how = 'left',
                on = 'Well')

#defining variables and conditions
conditions = [100, 10, 1, 0]
species = ['25', '27', '32', '35',
            '40', '41', '42', '49',
            '50', '26', '28', '29',
            '30', '31', '33', '34',
            '36', '37', '38', '39',
            '43', '44', '45', '46',
            '47', '48', '51', 'WT',
            'GFP', '-']
mutations = ['Blank','216/516','214/442','159/513','167/517','158/512',
             '169/516','237/439','238/516','156/513','161/516','215/520',
             '167/513','169/512','156/516','238/442','170/513','158/514',
             '167/516','191/513','159/512','191/517','215/524','169/510',
             '159/517','191/520','169/514','214/524','2TAG-sfGFP', 'WT']
time = full_data.iloc[0:41,0]
replicate = [1,2,3]
species = sorted(species)
conditions = sorted(conditions)
##setting up sliding window length of five points
window = 5

#setting up plots and subplots
fig, ax = plt.subplots(nrows = 6,
                       ncols = 5,
                       sharex = True,
                       sharey = True)
colors = plt.cm.Greens(np.linspace(0.3,0.9,len(conditions)))

##setting up dictionaries to store values
dict = {}
slope_dict = {}

#setting up loops to calculate rates
for enz in species:
    for conc in conditions:

        ###only look at data of one enz @ one ni conc
        mini_data = full_data.loc[(full_data['species'] == enz)&
            (full_data['Ni (uM)'] == conc)]

        ###initialize an empty slope variable for all slopes
        ### over a window of time
        allslope = []
        allslopestd = []

        ###set up sliding window
        for t in range(0,len(time[0:-(window-1)])):
            timepoints = time[t:t+window]
            timepoints = timepoints.tolist()

            temp_slope = []
            temp_rsquared = []
```

```
            for rep in replicate:

            ##initialize empty array for rfu values
                rfu = []

            ###for each point in the sliding window, find all rfu
            ###that correspondong to that time point
                for x in timepoints:
                    rfu_reps = mini_data['rfu'].loc[(mini_data['Time'] == x)&(mini_data['replicate'] == rep)]
                    rfu_reps = rfu_reps.tolist()
                    rfu.append(rfu_reps)

                ## change rfu from list of tuples to just a list
                rfu = [y[0] for y in rfu]

                ###after populating rfu for a window of time
                ###(timepoints), do regression on those points
                var = linregress(timepoints, rfu)
                slope = var[0]
                rsquared = var[2]*var[2]

                ###append those values to temp_slope,temp_rsquared
                temp_slope.append(slope)
                temp_rsquared.append(rsquared)

            ###after the loop over replicates, we have replicates of slopes
            ###for each window of time. now avg them together.
            avgslope = np.mean(temp_slope)
            stdevslope = np.std(temp_slope)

            ###store the avg slope in allslope, which captures slope over time
            allslope.append(avgslope)
            allslopestd.append(stdevslope)

        ##once all slopes over all time have been calculated, append that
        ##to dictionary to store the value
        dict[enz+str(conc)] = (allslope, allslopestd)

        ###find the max slope and its stdev over time period
        maxslope = max(dict[enz+str(conc)][0])
        maxindex = dict[enz+str(conc)][0].index(maxslope)
        maxslopestd = dict[enz+str(conc)][1][maxindex]

        ###store maxslope, stdev at maxslope in new dict
        slope_dict[enz+str(conc)] = (maxslope,maxslopestd)

###plotting
count1 = 0
count2 = 0
count3 = 0
for enz in species:
    for conc in conditions:
        ax[count1,count2].bar(str(conc),slope_dict[enz+str(conc)][0],     yerr     =     slope_dict[enz+str(conc)][1],color     =
colors[conditions.index(conc)])
        ax[count1,count2].set_title(mutations[count3], fontsize = 7)
    count2 = count2 + 1
    if count2 > 4:
        count1 = count1 + 1
        count2 = 0
    count3 = count3 + 1

for ax in fig.axes:
    plt.sca(ax)
    plt.xticks(rotation=45)

###adjustments on plots
fig.text(0.5,0.05, 'Ni (μM)', ha='center', va='center')
fig.text(0.05, 0.5, 'Rate (AU/min) at 30°C', va = 'center', rotation = 'vertical')
fig.subplots_adjust(left = 0.15, bottom = 0.13, wspace = 0.4, hspace = 0.8)
#fig.savefig('210603_rates_bar_Ni.pdf', bbox_inches='tight')
```

<u>High-throughput screening of luciferase mutants</u>

All Pluc variants, a WT Pluc, and 2TAG sfGFP were synthesized by CFPS, along with a sufficient volume of blank CFPS reactions as diluent. After reactions were complete, Pluc mutants were diluted to 1.1 µM with blank CFPS reactions. Assay mixtures were then set up by mixing 8.5 µL of Pluc-enriched extracts, 42.5 µL of 2X Assay Buffer (2 mM ATP, 20 mM Magnesium Sulfate, 25 mM HEPES pH 7.8), 8.5 µL of 0 – 1 mM Nickel(II) Sulfate, and 21.3 µL water. Enzymes were equilibrated on ice for 1 hour. During incubation on ice, 1 µL of 1.2 mM D-Luciferin (cat. L9504) in DMSO was spotted into a 384-well plate using the Echo 550. The assay mixture was then split into three aliquots. 19 µL of each aliquot was transferred into wells containing D-Luciferin with the Integra Viaflo. The reactions were mixed by pipetting, spun down, and placed into a BioTek

Synergy H1 Microplate reader. Measurements were taken for 1 h at room temperature. Maximum RLU values were then identified and plotted.

Kinetic analysis of luciferase mutants

$Pluc_{202/532}$, $Pluc_{108/508}$, WT Pluc, and blank CFPS reactions were set up as previously described. After CFPS reactions were complete, all Pluc enzymes were kept on ice until immediately before measurement due to rapid loss of activity at room temperature. All Pluc enzymes were diluted to 1.1 μM in blank CFPS reactions. Eight standards of luciferin, as well as a zero, ranging from 15 mM to 6.9 μM were made using three-fold serial dilutions in DMSO for a total of nine standards. 80 μL of Pluc enzymes were incubated with an equal volume of either water or 1M Nickel (II) Sulfate on ice for one hour. In separate tubes, Substrate Buffers containing 400 μL of 2X Assay Buffer, 200 μL of water, and 40 μL of a single luciferase standard were mixed and equilibrated to room temperature. After equilibration, 56 uL of Substrate Buffer was mixed with 14 uL of Pluc mixture, split into three 20 μL replicates in a 384-well plate, and immediately read for 5 min in a room-temperature BioTek Synergy H1 Microplate reader. The maximum RLU values were extracted from each set of standards for all Pluc enzymes. Using OriginPro, these RLU_{max} values and their corresponding D-Luciferin concentrations were fit to a Michaelis-Menten curve accounting for inhibition observed at higher substrate concentrations (Equation 1). The equation is as follows:

(Equation 1): $V = \frac{V_{\max}[S]}{K_M + [S]\left(1 + \frac{[S]}{K_i}\right)}$

Low-Throughput Procedures and Large-Scale Characterization

General Materials and Methods

Unless otherwise noted, all reagents were obtained from commercial suppliers and used without further purification. Aqueous solutions were prepared using Milli-Q water. PrimeSTAR Max DNA polymerase master mix was purchased from Takara Bio (Mountainview, CA). Restriction enzymes and CutSmart buffer were purchased from New England Biolabs, Inc (Ipswitch, MA). Vivaspin 20 ultrafiltration units (20 mL volume, 30 kDa cutoff) from Sartorius (Goettingen, Germany) were used to concentrate proteins and perform diafiltration. QIAquick Gel Extraction Kit and QIAprep Spin Miniprep Kit were purchased from QIAGEN Inc. (Valencia, CA) and used according to the manufacturer's instructions. Zeba desalting columns (2 mL, 30 kDa cutoff) were purchased from Fisher Scientific and used to prepare samples for analysis by intact protein ESI-MS. WHEATON 0.2 μm nylon syringe filters were purchased from DWK Life Sciences, LLC (Millville, NJ). BpyAla was either purchased from AmBeed (Arlington Hts, IL) and used without further purification or prepared according to protocol described above. Oligonucleotide primers were purchased from Sigma Aldrich (St. Louis, MO). pEVOL-BpyRS was generously provided by Peter Schultz of the Scripps Research Institute, CA. Luria broth (LB) and 2XYT broth were purchased from Research Products International, Corp (Mt. Prospect, IL). LB agar was purchased from BD (Franklin Lakes, NJ). Kanamycin monosulfate was purchased from Alfa Aesar (Tewksbury, MA) and prepared as a 50 mg/mL solution in water. Chloramphenicol (prepared as a 25 mg/mL solution in ethanol), isopropyl β-D-1-thiogalactopyranoside (IPTG, prepared as a 1 M solution in water), and L-arabinose (prepared as a 20% w/v solution in water) were purchased from Chem-Impex International (Wood Dale, IL). *E. coli* BL21-Gold (DE3) cells were purchased from Agilent (Santa Clara, CA) and used to prepare electrocompetent cells. DNA Clean and Concentrator kit was purchased from Zymo Research (Irvine, CA) and used to purify DNA after PCR reactions and restriction digests according to the manufacturer's instructions. Ammonium iron(II) sulfate hexahydrate was purchased from ACROS Organics (Fair Lawn, NJ). Nickel chloride (anhydrous)

was purchased from Strem Chemicals (Newburyport, MA). 1,10-phenathroline was purchased from Aldrich Chemical Co Inc (Milwaukee, WI). Tetrasodium EDTA and DMSO were purchased from Fisher Scientific (Waltham, MA). HisPur Ni-NTA resin and Pierce Coomassie (Bradford) Protein Assay Kit were purchased from Thermo-Fisher Scientific (Waltham, MA). Z-Ala-Pro-*p*NA was purchased from Bachem (Bubendorf, Switzerland).

All gene sequences were confirmed by DNA sequencing through QuintaraBio (Boston, MA). Electroporation was carried out on a Bio-Rad MicroPulser™ using method Ec2. Aqueous media was prepared using Milli-Q water and sterilized either by autoclaving or by filtration. Antibiotics were prepared as 1,000x stocks and stored at -20 °C. Final concentrations for antibiotics used were: Kanamycin at 50 µg/mL and Chloramphenicol at 25 µg/mL. *E. coli* cultures were handled using sterile technique under open flame. DNA was quantified by absorbance at 260 nm and purity was assessed by ratio of absorbance at 260nm/280nm using a Tecan Infinite M200 Pro plate reader with a NanoQuant plate. Protein concentrations were measured using the Pierce Coomassie (Bradford) Protein Assay Kit with a standard curve generated from standard BSA control samples and protein stocks were then flash frozen with liquid N_2 and stored at -80 °C until use. Standard molecular cloning procedures were followed. Gels were imaged with an Alpha Innotech AlphaImager EP. FPLC was performed on a GE AKTA purifier equipped with a UPC-900 detector (monitoring absorbance at 280 nm) and P-900 pumps. Intact protein mass spectrometry was performed using a Waters Synapt G2S HDMS using a C18 column. Protein samples were desalted using manufacturer specifications before MS analysis. UV-Vis spectroscopy was performed using a Cary 5000 UV-Vis-NIR spectrophotometer at room temperature after blanking with solution containing just water using 10 mm pathlength quartz cuvettes. Circular dichroism (CD) spectra were obtained at room temperature on a JASCO J-1500 CD Spectrometer using 10 mm pathlength quartz cuvettes. Kinetic assays were performed on a Varian Cary 100 Bio UV-Vis spectrophotometer using 10 mm pathlength quartz cuvettes. ICP-MS was performed using an Agilent 8800 QQQ ICP-MS.

Cloning of TAG (amber stop codon) POP variants

pET28a plasmid vector containing a codon optimized gene for *Pyrococcus furiosus* prolyl oligopeptidase[6] (POP) (cloned using NcoI and XhoI restriction sites upstream of a C-terminal hexa-histidine tag for Ni-NTA affinity chromatography) was previously prepared and used for cloning of BpyAla variants. Variants were constructed using Quikchange mutagenesis to site-specifically introduce amber stop codons (TAG). The following PCR conditions were used: 25 µL PrimeSTAR Max premix (2X), 0.8 µM forward primer, 0.8 µM reverse primer, >50 ng template plasmid, adjusted to a final volume of 50 µL with sterile DNase- and RNase-free water.

Thermal cycler was programmed as:
1. 98 °C -120 seconds
2. 98 °C - 10 seconds
3. 55 °C - 15 seconds
4. 72 °C - 150 seconds
5. Repeat cycles from steps #2 to #4 30 times
6. 72 °C - 5 mins
7. 4 °C – hold

Supplementary Table 6. Primers used for POP TAG variant generation.

**Primer name**	**Primer Sequence**
167TAG_F	5'- GAT GGT GTC TAG CCG CCG GCA GC -3'
167TAG_R	5'- GCT GCC GGC GGC TAG ACA CCA TC -3'
169TAG_F	5'- GTC AAT CCG TAG GCA GCA CGT ATG TTT TGG -3'
169TAG_R	5'- CGT GCT GCC TAC GGA TTG ACA CCA TCC -3'
510TAG_F	5'- CTG CGT TTT TAG AAA CTG TAT ATT GGC TCT G -3'
510TAG_R	5'- CAG TTT CTA AAA ACG CAG CAT ATC GAT CAC -3'
512TAG_F	5'- GTT TTC ATA AAT AGT ATA TTG GCT CTG TGT GG -3'
512TAG_R	5'- CAA TAT ACT ATT TAT GAA AAC GCA GCA TAT C -3'
513TAG_F	5'- CAT AAA CTG TAG ATT GGC TCT GTG TGG ATT C -3'
513TAG_R	5'- CAG AGC CAA TCT ACA GTT TAT GAA AAC GCA G -3'
516TAG_F	5'- GTA TAT TGG CTA GGT GTG GAT TCC GGA ATA C -3'
516TAG_R	5'- GAA TCC ACA CCT AGC CAA TAT ACA GTT TAT G -3'
517TAG_F	5'- GGC TCT TAG TGG ATT CCG AAT ACG GTA AC -3'
517TAG_R	5'- CGG AAT CCA CTA AGA GCC AAT ATA CAG TTT ATG -3'
T7-Promoter	5'- TAA TAC GAC TCA CTA TAG GG -3'
T7-Terminal	5'- GCT AGT TAT TGC TCA GCG G -3'

An analytical gel (1% agarose) of the PCR reaction (5 µL) was run to confirm successful amplification of the product. The PCR product was then digested with DpnI to degrade template DNA for 2 hours at 37 °C and then stored at 4 °C until further use. The following digestion reaction conditions were used: 45 µL PCR product, 10 µL CutSmart buffer (10X), 2 µL DpnI, and 43 µL sterile DNase- and RNase-free water. The reaction mixture was cleaned using DNA purification kits and transformed into electrocompetent E. coli BL21-Gold (DE3) cells. Cells were recovered in LB medium for 1 hour at 37 °C (250 rpm) before spreading on LB agar plates with antibiotics (0.05 mg/mL kanamycin, 0.025 mg/mL chloramphenicol) and incubating at 37 °C overnight. To verify the genotype, individual colonies were inoculated in LB media (with 0.05 mg/mL kanamycin) and grown overnight at 37 °C (250 rpm). Plasmids from these overnight grown cultures were isolated using QiaPrep kits) and sequenced by QuintaraBio (Boston, MA). T7-Promoter and T7-Terminal primers (provided by QuintaraBio) were used for sequencing reactions. Nucleotide sequences for all the primers used above are summarized in Supplementary Table 6. Once the genotype was confirmed with sequencing, the plasmid harboring the POP gene was transformed into electrocompetent E. coli BL21-Gold (DE3) cells containing pEVOL-BpyRS plasmid in the same manner described above except that selection was performed in the presence of both kanamycin (0.05 mg/mL) and chloramphenicol (0.02 mg/mL). Glycerol stocks of cells were prepared with 0.5 mL overnight culture (inoculated from a single colony) and 0.5 mL sterile 50% (v/v) glycerol and stored at -80 °C.

Large-Scale Protein Expression

Protein for all applications was expressed in the same manner as described here. 5 mL of LB media (with 0.05 mg/mL kanamycin and 0.02 mg/mL chloramphenicol) was inoculated from the appropriate glycerol stock and grown overnight at 37 °C (250 rpm). The following day, 5 mL of the O/N culture was used to inoculate 500 mL of 2XYT media with 0.05 mg/mL kanamycin and 0.02 mg/mL chloramphenicol) in a 2.8L Fernbach flask. The culture was grown at 37 °C (250 rpm) until the OD_{600} was between 0.6 and 1.0. To induce overexpression from the pEVOL plasmids (required for incorporation of ncAAs), R,S-(2,2'-Bipyridin-5-yl)alanine (1-2 mM final concentration in 500 mL culture) dissolved in 2.5 mL 20% (w/v) L-arabinose was added to the culture. To induce

overexpression of POP, IPTG (1 mM final concentration) was added and the cells were allowed to grow overnight at 37 °C (250 rpm). The cells were harvested by centrifugation at 3,600 rpm (4 °C) for 30 minutes. The supernatant was discarded, and the pellet was resuspended in 50 mL equilibration buffer (20 mM Na_2HPO_4, 300 mM NaCl, 10 mM imidazole, pH 7.4). The cells were split into 25 mL aliquots (in 50 mL conical tubes) and then stored at -80 °C until lysis.

Steady-State Kinetic Assays

Protein Purification

Cells were lysed by sonication on ice with a cylindrical horn (40 W amplitude, 30 second bursts, 5 minutes 'on' time, 10 minutes total). The cells were then heated at 85 °C for 30 minutes as an initial purification step (since POP is hyperthermophilic), and the lysate was clarified by centrifugation at 12,000 rpm (4 °C) for 30 minutes. The soluble lysate fraction (supernatant) was immediately decanted into a new conical tube. The lysate was loaded onto Ni-NTA resin (5 mL) and the flow-through was discarded. The resin was washed with 10 CV of wash buffer (20 mM Na_2HPO_4, 300 mM NaCl, 20 mM imidazole, pH 7.4). The protein was eluted using 10 CV of elution buffer (20 mM Na_2HPO_4, 300 mM NaCl, 250 mM imidazole, pH 7.4).

For steady-state kinetic assays with variants $POP_{159/517}$, $POP_{167/513}$, $POP_{169/510}$, and $POP_{169/512}$, protein samples eluted from Ni-NTA resin were then concentrated to a volume less than 1 mL. The samples were heated with 100 mM EDTA (pH 8.0) at 55 °C for 5 minutes. Diafiltration was then performed with MQ H_2O and samples were used for kinetics without any further purification.

For steady-state kinetic assays with variants POP WT, POP_{167}, POP_{517}, and $POP_{167./517}$, samples were concentrated to a volume less than 1 mL after IMAC purification. The samples were then incubated with 100 mM EDTA (pH 8.0) at 37 °C for 1 hour. Samples were purified by FPLC using a size-exclusion column (SEC). A HiLoad 16/600 Superdex 200 column (GE Life Sciences) was equilibrated 2 CVs of the mobile phase (sterile filtered 150 mM NaCl solution). The flow rate was set to 1 mL/min and fractions were manually collected (the proteins of interest eluted between 75-83 mL). Samples were then concentrated and diafiltration was performed with MQ H_2O.

Steady-State Kinetic Assays

The hydrolysis of benzyloxycarbonyl-alanyl-prolyl-p-nitroanilide (Z-Ala-Pro-pNA) was monitored by spectrophotometry at 85 °C.[27] The initial rate of reaction was determined by measuring the production of the yellow product, p-nitroaniline (pNA) which has a calculated molar extinction coefficient of 7,126 M^{-1} cm^{-1} at 410 nm. 900 µL of master mix solution containing buffer (33 mM HEPES, pH 7.4 and 889 mM NaCl), enzyme, and either EDTA (1.11 mM, pH 8.0) or $NiCl_2$ (5.56 µM) was added to a quartz cuvette and incubated at 85 °C in the spectrophotometer for 2 minutes. To initiate the reaction, 1-100 µL of the substrate Z-Ala-Pro-pNA (10 mM in DMSO) and 0-99 µL of DMSO (so that the final concentration of DMSO was 10% v/v) were added to the cuvette and the solution was mixed by pipetting up and down. Final concentrations of components in the reaction were as follows: 30 mM HEPES (pH 7.4), 800 mM NaCl, 10-30 nM enzyme, either 5 µM $NiCl_2$ or 1 mM EDTA, 0.01-1.00 mM Z-Ala-Pro-pNA, and 10% (v/v) DMSO. The reaction was monitored with absorbance measurements (at 410 nm) every 6 seconds for 1 minute. Initial rates were determined by converting absorbance values to concentrations using the molar extinction coefficient in Excel. Initial rates were plotted versus substrate concentration in Origin Pro and non-linear fitting of the data with the Michaelis-Menten equation (Equation 2) was performed. All data were collected in triplicate and the standard deviation is represented by errors bars.

(Equation 2): $V = \frac{V_{\max}[S]}{K_M + [S]}$

Activity Switching Assays

Protein Purification

Cells were lysed by sonication on ice with a cylindrical horn (40 W amplitude, 30 second bursts, 5 minutes 'on' time, 10 minutes total). The cells were then heated at 85 °C for 30 minutes as an initial purification step (since POP is hyperthermophilic), and the lysate was clarified by centrifugation at 12,000 rpm (4 °C) for 30 minutes. The soluble lysate fraction (supernatant) was immediately decanted into a new conical tube. The lysate was loaded onto Ni-NTA resin (5 mL) and the flow-through was discarded. The resin was washed with 10 CV of wash buffer (20 mM Na_2HPO_4, 300 mM NaCl, 20 mM imidazole, pH 7.4). The protein was eluted using 10 CV of elution buffer (20 mM Na_2HPO_4, 300 mM NaCl, 250 mM imidazole, pH 7.4).

Protein samples eluted from Ni-NTA resin were then concentrated to a volume less than 1 mL. The samples were heated with 100 mM EDTA (pH 8.0) at 55 °C for 5 minutes. Diafiltration was then performed with MQ H_2O and samples were used for kinetics without any further purification.

Switching Assay

The switching of activity between 'on' and 'off' states was performed using a modified version of the kinetic assays described above (see Steady-State Kinetic Assays above). 500 µL of a 1 µM stock of protein (in MQ H_2O) was incubated at 55 °C with shaking (750 rpm) for the entirety of the assay using a Thermo Scientific™ Thermal Mixer (with Blocks). For the first round, 1 µL of $NiCl_2$ solution (10 mM) was added to the protein stock and the sample was incubated for 2 minutes. An aliquot of protein was removed from the stock and added to a 10 mm pathlength quartz cuvette containing buffer. The amount of protein added to the reaction buffer was adjusted throughout the assay based on the changing concentration due to additions of EDTA/$NiCl_2$ (Supplementary Table 7). The cuvette was then incubated at 85 °C for 2 minutes. At the same time, 2 µL of EDTA (10 mM, pH 8.0) was added to the protein stock for the second round and a 2-minute incubation was started. After incubating the cuvette, the reaction was initiated by addition of 100 µL of Z-Ala-Pro-pNA (10 mM in DMSO) and the solution was mixed by pipetting up and down several times. The reaction was monitored by following the formation of *p*NA with absorbance measurements (at 410 nm) every 6 seconds for 1 minute. Initial rates were determined by converting absorbance values over time to concentrations of time using the molar extinction coefficient (ε_{410} = 7,126 M^{-1} cm^{-1}) in Excel. Simultaneously, an aliquot of protein (20.1 µL) was removed from the protein stock solution and added to a cuvette containing buffer and was incubated at 85 °C for 2 minutes. This cycle was repeated in the same manner by alternating additions of $NiCl_2$ and EDTA. Final concentrations of components in the reaction were as follows: 30 mM HEPES (pH 7.4), 100 mM NaCl, 20 nM enzyme, 0.2-30 mM $NiCl_2$, 0-41 mM EDTA, 1.00 mM Z-Ala-Pro-pNA, and 10% (v/v) DMSO. All data were collected in triplicate and averaged. Relative rates were determined by dividing each average initial rate by the maximum average rate collected for that data set so that the highest relative rate was 1. The relative initial rates were plotted versus round to assess the switching of the systems. Error bars represent propagated standard deviations.

Supplementary Table 7. Conditions for switching assay.

	**Added to Protein Stock**		**Added to Reaction**			
**Round Number**	**EDTA (10 mM)**	**$NiCl_2$ (10 mM)**	**Enzyme**	**MQ H_2O**	**60 mM HEPES (pH 7.4) + 200 mM NaCl**	**Substrate (10 mM)**
1	0 µL	1 µL	20 µL	380 µL	500 µL	100 µL
2	2 µL	0 µL	20.1 µL	380 µL	500 µL	100 µL
3	0 µL	3 µL	20.3 µL	380 µL	500 µL	100 µL
4	4 µL	0 µL	20.4 µL	380 µL	500 µL	100 µL
5	0 µL	5 µL	20.7 µL	379 µL	500 µL	100 µL
6	6 µL	0 µL	21 µL	379 µL	500 µL	100 µL
7	0 µL	7 µL	21.3 µL	379 µL	500 µL	100 µL
8	8 µL	0 µL	21.8 µL	378 µL	500 µL	100 µL
9	0 µL	9 µL	22.3 µL	378 µL	500 µL	100 µL
10	10 µL	0 µL	22.9 µL	377 µL	500 µL	100 µL
11	0 µL	11 µL	23.7 µL	376 µL	500 µL	100 µL
12	12 µL	0 µL	24.5 µL	375 µL	500 µL	100 µL
13	0 µL	13 µL	25.5 µL	374 µL	500 µL	100 µL
14	14 µL	0 µL	26.7 µL	373 µL	500 µL	100 µL
15	0 µL	15 µL	28.1 µL	372 µL	500 µL	100 µL
16	16 µL	0 µL	29.7 µL	370 µL	500 µL	100 µL
17	0 µL	17 µL	31.6 µL	368 µL	500 µL	100 µL
18	18 µL	0 µL	33.8 µL	366 µL	500 µL	100 µL
19	0 µL	19 µL	36.5 µL	363 µL	500 µL	100 µL
20	20 µL	0 µL	39.8 µL	360 µL	500 µL	100 µL
21	0 µL	21 µL	44 µL	356 µL	500 µL	100 µL
22	22 µL	0 µL	49.5 µL	350 µL	500 µL	100 µL
23	0 µL	23 µL	57.2 µL	343 µL	500 µL	100 µL
24	24 µL	0 µL	69.2 µL	331 µL	500 µL	100 µL

Physical Characterization of POP Variants

Protein Purification for Intact Protein ESI-MS (EDTA-treatment)
Cells were lysed by sonication on ice with a cylindrical horn (40 W amplitude, 30 second bursts, 5 minutes 'on' time, 10 minutes total). The cells were then heated at 85 °C for 30 minutes as an initial purification step (since POP is hyperthermophilic), and the lysate was clarified by centrifugation at 12,000 rpm (4 °C) for 30 minutes. The soluble lysate fraction (supernatant) was immediately decanted into a new conical tube. The lysate was loaded onto Ni-NTA resin (5 mL) and the flow-through was discarded. The resin was washed with 10 CV of wash buffer (20 mM Na_2HPO_4, 300 mM NaCl, 20 mM imidazole, pH 7.4). The protein was eluted using 10 CV of elution buffer (20 mM Na_2HPO_4, 300 mM NaCl, 250 mM imidazole, pH 7.4).

Samples were concentrated to a volume less than 1 mL after IMAC purification. The samples were then incubated with 100 mM EDTA (pH 8.0) at 37 °C for 1 hour. Samples were purified by FPLC using a size-exclusion column (SEC). A HiLoad 16/600 Superdex 200 column (GE Life Sciences) was equilibrated 2 CVs of the mobile phase (sterile filtered 150 mM NaCl solution). The flow rate was set to 1 mL/min and fractions were manually collected (the proteins of interest eluted between 75-83 mL). Samples were then concentrated and diafiltration was performed with MQ H_2O.

Intact Protein ESI-MS for EDTA- and Ni^{2+}-treated Samples
POP_{WT}, POP_{167}, POP_{517}, and $POP_{167/517}$ samples were adjusted to 3 µM in either 150 µL of 150 mM EDTA or 0.75 mM $NiCl_2$. Samples were heated at 85 °C (750 rpm) in a Thermo Scientific™ Thermal Mixer (with Blocks) for 2 minutes. Protein samples were then desalted using Zeba desalting columns (according to the manufacturer's instructions), filtered through 0.2 µm nylon syringe filters, and used for mass spectrometric analysis.

Intact protein mass spectrometry was performed using a Waters Synapt G2S HDMS using a C18 column. A 10-minute LC method (A: H_2O with 0.1% formic acid, B = acetonitrile with 0.1% formic acid) with a linear gradient from 95% A to 1% A over 6 minutes followed by a 4-minute flush at 95% A was used with the mass spectrometer recording between 400-2000 Da, providing an LC trace like the representative ones in Supplementary Figure 19 (protein retention time = 4.24 mins). Deconvolution of the mass spectrum was performed using either a 700-900 M/Z window (for all samples) or a 1100-1500 M/Z window (performed for $POP_{167/517}$ that was treated with $NiCl_2$ to analyze the second feature unique to that sample) with a deconvoluted mass range of 70-75 kDa.

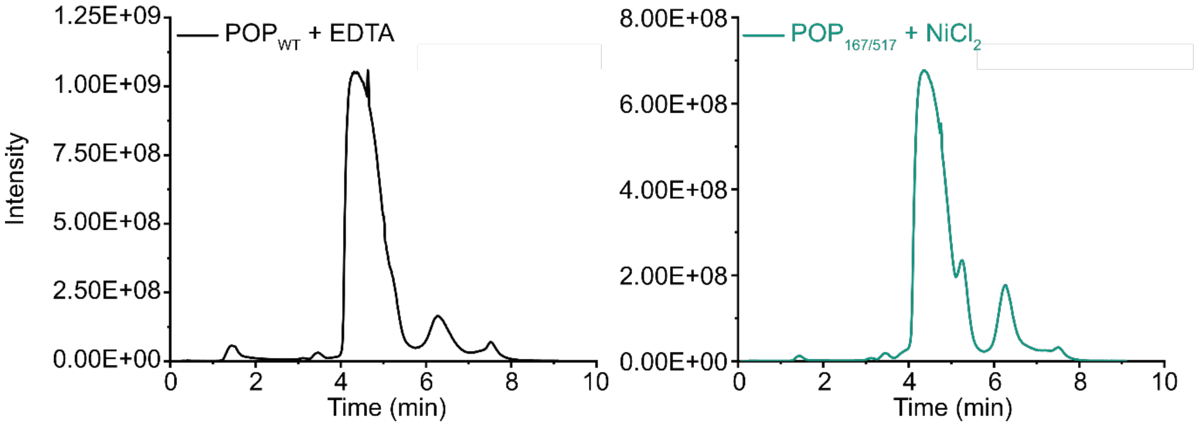

Supplementary Figure 19. Representative LC traces of purified POP variants analyzed by LC-MS.
The chromatograms obtained from LC separation of different POP samples were nearly identical. Shown here, are selected LC traces for POP_{WT} that was treated with EDTA (left) and $POP_{167/517}$ treated with $NiCl_2$ (right). In all cases, POP elutes at approximately 4.2 minutes.

Protein Purification for UV-Vis, CD, ICP-MS, and Intact Protein MS (1,10-phenathroline-treatment)
Cells were lysed by sonication on ice with a cylindrical horn (40 W amplitude, 30 second bursts, 5 minutes 'on' time, 10 minutes total). The cells were then heated at 85 °C for 30 minutes as an initial purification step (since POP is hyperthermophilic), and the lysate was clarified by centrifugation at 12,000 rpm (4 °C) for 30 minutes. The soluble lysate fraction (supernatant) was immediately decanted into a new conical tube. The lysate was loaded onto Ni-NTA resin (5 mL) and the flow-through was discarded. The resin was washed with 10 CV of wash buffer (20 mM Na_2HPO_4, 300 mM NaCl, 20 mM imidazole, pH 7.4). The protein was eluted using 10 CV of elution buffer (20 mM Na_2HPO_4, 300 mM NaCl, 250 mM imidazole, pH 7.4).

Protein samples eluted from Ni-NTA resin were then concentrated to a volume less than 1 mL. For the purposes of UV-Vis and CD, it was critical to remove all traces of Fe(II) as $Fe(bpy)_n^{2+}$ species have strong electronic transitions in the UV and visible light regions[2]. Even though we

observed the apo mass by intact protein ESI-MS for EDTA treated samples, suggesting the removal of most metals, we still observed a slight pink color in variant $POP_{167/517}$, due to the presence of iron. Thus, we switched to 1,10-phenanthroline as it has previously been established to be effective at sequestering Fe from affinity binding sites within proteins[2]. Samples were buffer exchanged into buffer containing 50 mM Tris (pH 7.4) and 150 mM NaCl by diafiltration and concentrated to less than 1 mL. Protein (75 μM) was mixed with 1,10-phenanthroline to give a final chelator concentration of 5 mM. Samples were incubated in a 65 °C water bath for 1 hour, before immediately purifying by SEC. A HiLoad 16/600 Superdex 200 column (GE Life Sciences) was equilibrated 2 CVs of the mobile phase (sterile filtered buffer containing 50 mM EDTA (pH 8.0) and 150 mM NaCl solution). The flow rate was set to 1 mL/min and fractions were manually collected (the proteins of interest eluted between 75-83 mL). Samples were then concentrated and diafiltration was performed with MQ H_2O. Prior to treatment with 1,10-phenanthroline, $POP_{167/517}$ was notably pink. After treatment with the chelating agent, the color of the solution changed slightly to a reddish-brown color, indicative of formation of $Fe(phen)_3^{2+}$. In addition to elution of the desired protein, two highly absorbing species eluted afterwards including a red-color small molecule (likely the iron phenanthroline complex) and a colorless small molecule (likely excess phenanthroline). Representative chromatograms are shown in Supplementary Figure 20.

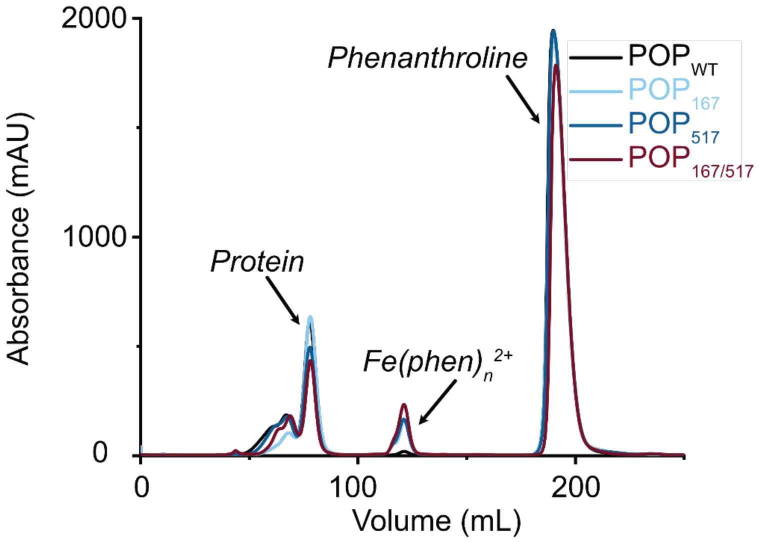

Supplementary Figure 20. SEC chromatograms of POP variants after treatment with phenanthroline.

Size-exclusion chromatography of POP_{WT}, POP_{167}, POP_{517}, and $POP_{167/517}$ was performed after treatment with 1,10-phenathroline to remove Fe from protein samples. Absorbance at 280 nm versus volume is shown. The desired apo proteins eluted from 75-83 mL as colorless solutions. A colored species eluted next, around 120 mL, that is likely an $Fe(phen)_n$ complex due to its characteristic red-orange color. Finally, a highly absorbing and colorless molecule eluted around 180 mL, that is likely excess phenanthroline.

Metalation with Fe^{2+}

Protein samples were diluted to 50 μM in a total volume of 250 μL with 250 μM $(NH_4)_2Fe(SO_4)_2{\cdot}6H_2O$ in MQ H_2O (5 equivalents). Samples were incubated at 65 °C for 15 minutes and diafiltration was then performed with MQ H_2O to remove residual Fe(II).

UV-Vis Spectroscopy
Samples were diluted to 50 µM with MQ H_2O and UV-Vis spectra were collected at room temperature. A spectrum of a blank solution was collected first, and then subtracted from all protein spectra. 1 scan from 800-200 nm (1 nm step) was performed in a 10 mm pathlength quartz cuvette.

Circular Dichroism
Samples were diluted to either 50 or 5 µM with MQ H_2O and CD spectra were collected at 25 °C. 3 accumulations from 700-170 nm were performed in a 10 mm pathlength quartz cuvette. The following parameters were utilized: 1.0 nm band width, 100 nm/min scan rate, and 0.1 nm data pitch. 50 µM was required to clearly see the features at ~300 nm in metalated BpyAla POP variants, but at these high concentrations, the absorbance at wavelengths >280 nm was too high, leading to noisy data. To visualize the secondary structures of the proteins, samples were diluted to 5 µM, where a characteristic peak for POP was observed around 220 nm (Supplementary Fig. 21), matching previously reported spectra[28].

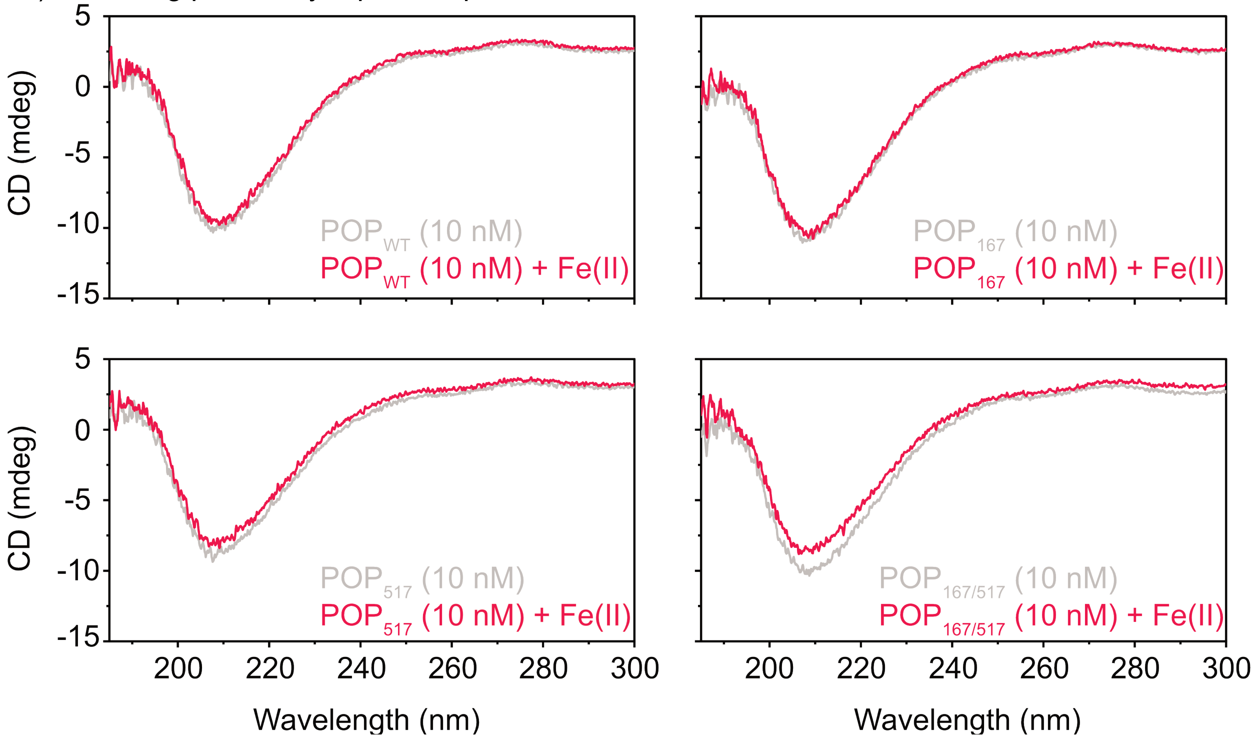

Supplementary Figure 21. CD spectra of various dilute POP samples.
CD spectra were collected of POP_{WT}, POP_{167}, POP_{517}, and $POP_{167/517}$ (all at 10 nM) in the absence or presence of Fe(II). At the higher concentrations (50 µM) necessary to visualize certain features associated with the metalloprotein (i.e. Cotton effects near 300 nm), the signal was too high to clearly observe secondary structural elements of the protein present in the far-UV region, thus we measured spectra at lower concentrations as well. A deep peak around 210 nm, characteristic of folded POP was observed.[28]

ICP-MS
Protein samples were diluted 100-fold into a final volume of 3 mL with 2.5% HNO_3 (Sigma) using Pure Plus Internal Standard Mix (100 ppb, PerkinElmer) as an internal standard. Samples were analyzed using an Agilent 8800 QQQ ICP-MS operating in hydrogen (^{55}Mn, ^{56}Fe, ^{59}Co, ^{60}Ni

detection) or helium (^{63}Cu, ^{66}Zn detection) as collision gases to remove possible interferences with ^{45}Sc or ^{72}Ge as the internal reference. Unknown sample metal concentrations were calculated from a standard curve of all analyzed metals made from Pure Plus Multi-Element Calibration Standard 3 (0.5-100 ppb, PerkinElmer).

Supplementary Table 8. ICP-MS data of POP variants before and after treatment with EDTA or 1,10-phenathroline.

Index	Variant	Condition	Mn (µM)	Fe (µM)	Co (µM)	Ni (µM)	Cu (µM)	Zn (µM)
1	POP_{WT}	Pre-Treatment	0.0	0.3	0.0	2.1	0.4	-0.5
2	POP_{WT}	Post-EDTA	0.1	0.6	0.5	0.3	3.1	-1.2
3	POP_{WT}	Post-Phen	0.0	-0.4	0.0	0.0	-0.1	-2.4
4	POP_{167}	Pre-Treatment	0.0	0.5	0.0	15.4	-0.1	-2.0
5	POP_{167}	Post-EDTA	0.0	0.4	0.2	0.2	0.2	-5.6
6	POP_{167}	Post-Phen	0.0	0.2	0.0	0.0	0.2	-2.5
7	POP_{517}	Pre-Treatment	0.0	4.6	0.0	18.9	-0.1	-1.7
8	POP_{517}	Post-EDTA	0.0	1.8	0.3	0.7	0.1	-4.8
9	POP_{517}	Post-Phen	0.0	-0.1	0.0	0.2	0.0	-2.7
10	$POP_{167/517}$	Pre-Treatment	0.0	7.8	0.0	18.2	-0.2	-1.1
11	$POP_{167/517}$	Post-EDTA	0.1	5.2	0.5	0.7	0.6	-5.5
12	$POP_{167/517}$	Post-Phen	0.0	1.1	0.0	0.4	-0.4	-2.9
13	Blank	NA	0.0	0.1	0.0	0.0	-0.1	0.0

Intact Protein ESI-MS for Fe^{2+}-treated Samples

Protein samples treated with Fe(II) were adjusted to 3 µM in 150 µL of MQ H_2O. Samples were then desalted using Zeba desalting columns (according to the manufacturer's instructions), filtered through 0.2 µm nylon syringe filters, and used for mass spectrometric analysis.

Intact protein mass spectrometry was performed using a Waters Synapt G2S HDMS using a C18 column. Intact protein mass spectrometry was used to analyze samples. A 10-minute LC method (A: H_2O with 0.1% formic acid, B = acetonitrile with 0.1% formic acid) with a linear gradient from 95% A to 1% A over 6 minutes followed by a 4-minute flush at 95% A was used with the mass spectrometer recording between 400-2000 Da (protein retention time = 4.24 mins). Deconvolution of the mass spectrum was performed using either a 700-900 M/Z window (for all samples) or a 1100-1500 M/Z window (performed for $POP_{167/517}$ that was treated with $(NH_4)_2Fe(SO_4)_2 \cdot 6H_2O$ to analyze the second feature unique to that sample) with a deconvoluted mass range of 70-75 kDa.

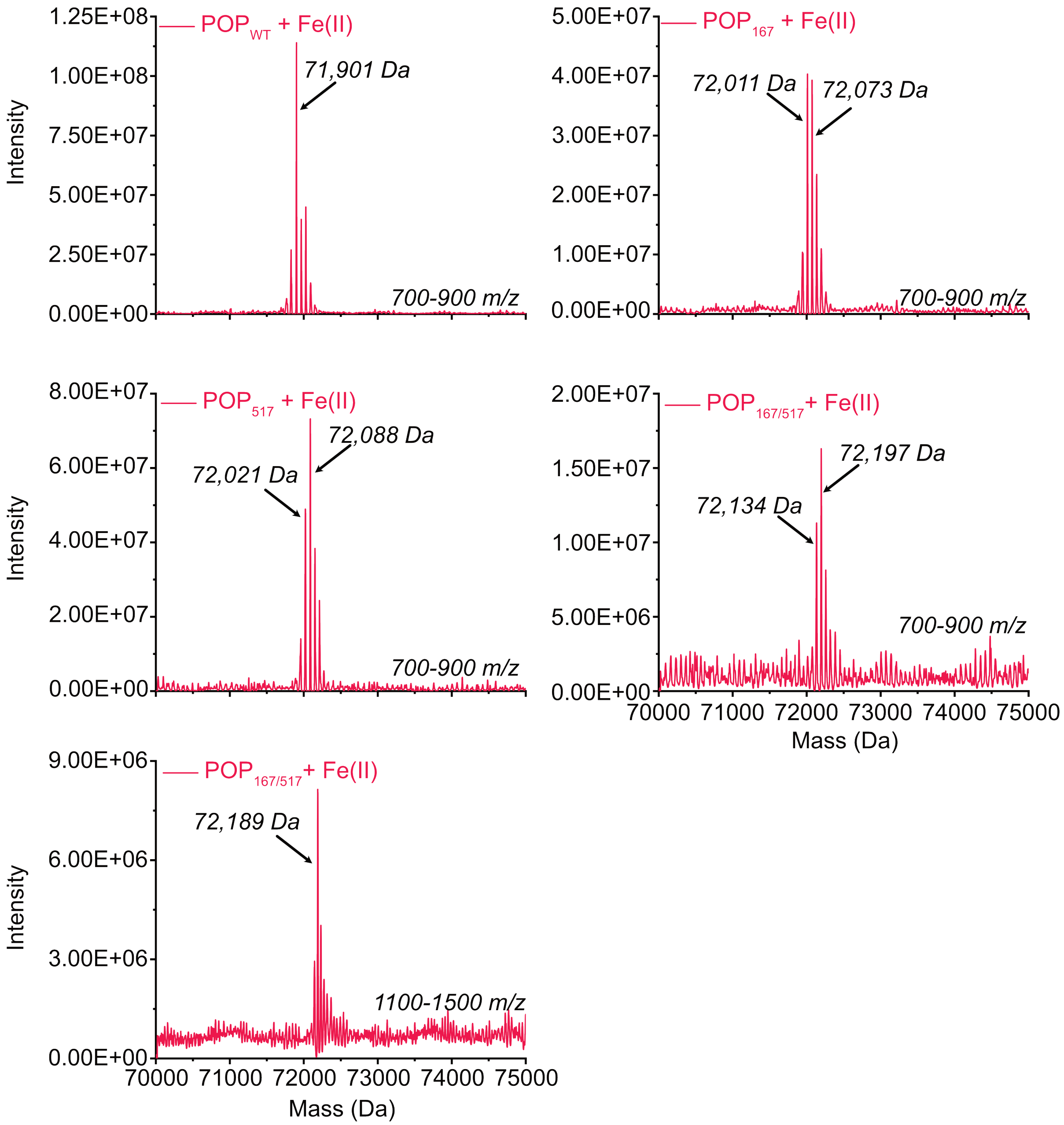

Supplementary Figure 22. Deconvoluted MS data for POP variants treated with Fe(II). Deconvoluted MS data for POP_{WT}, POP_{167}, POP_{517}, and $POP_{167/517}$ after treatment of the apo proteins with 5 equivalents of $(NH_4)_2Fe(SO_4)_2{\cdot}6H_2O$ and subsequent removal of residual Fe(II). Data was deconvoluted using 700-900 m/z and 1,100-1,500 m/z windows (only $POP_{167/517}$ was deconvoluted with both windows due to presence of high m/z feature; other variants were only deconvoluted with the lower m/z window) and the masses of the most intense from each sample are highlighted. Masses corresponding to the apo proteins were found in all MS from deconvolution with a 700-900 m/z window. Single metalation was observed for BpyAla POP variants in all deconvoluted spectra. However, the deconvoluted MS of $POP_{167/517}$ with the 1,100-1,500 m/z window showed only the single-metalated species, in contrast with the single BpyAla variants where both metalated and apo masses were prevalent.

Uncropped Gel Images

For Supplementary Figure 2:

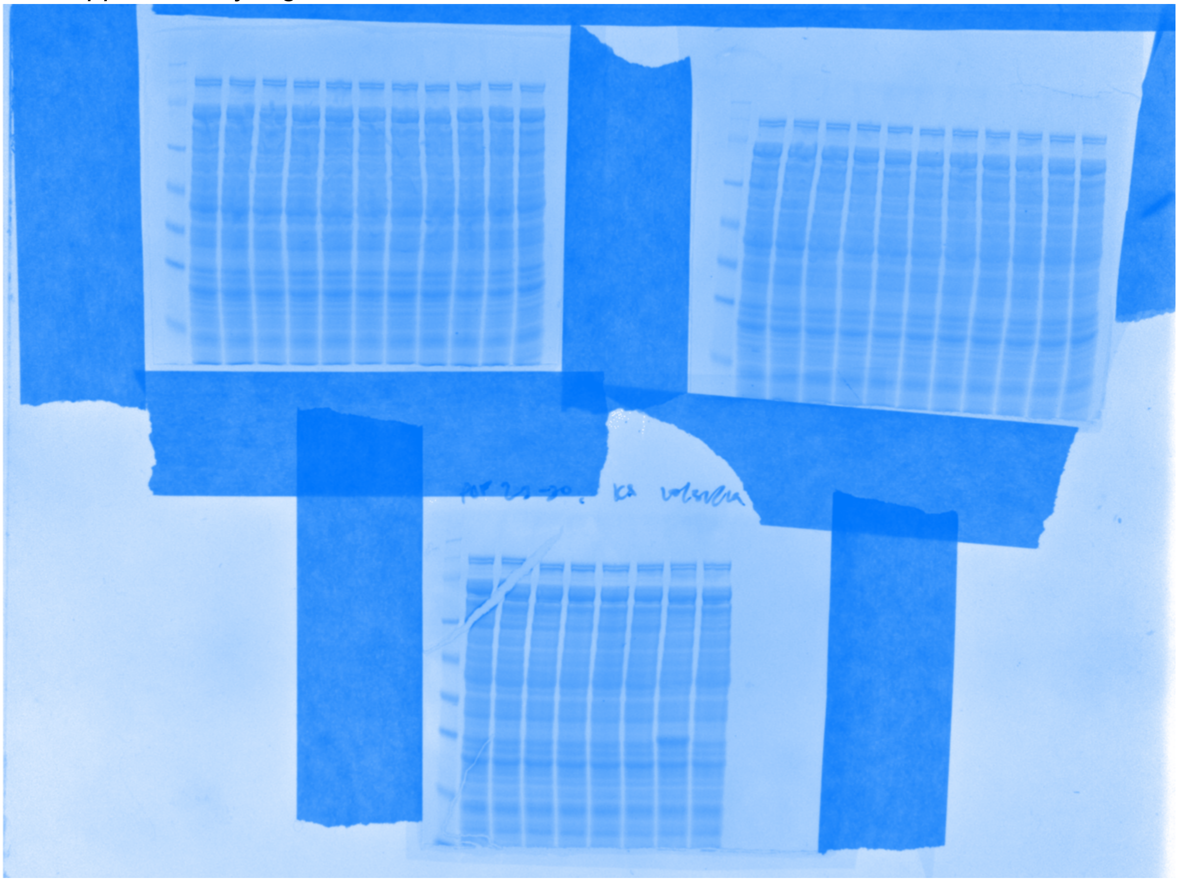

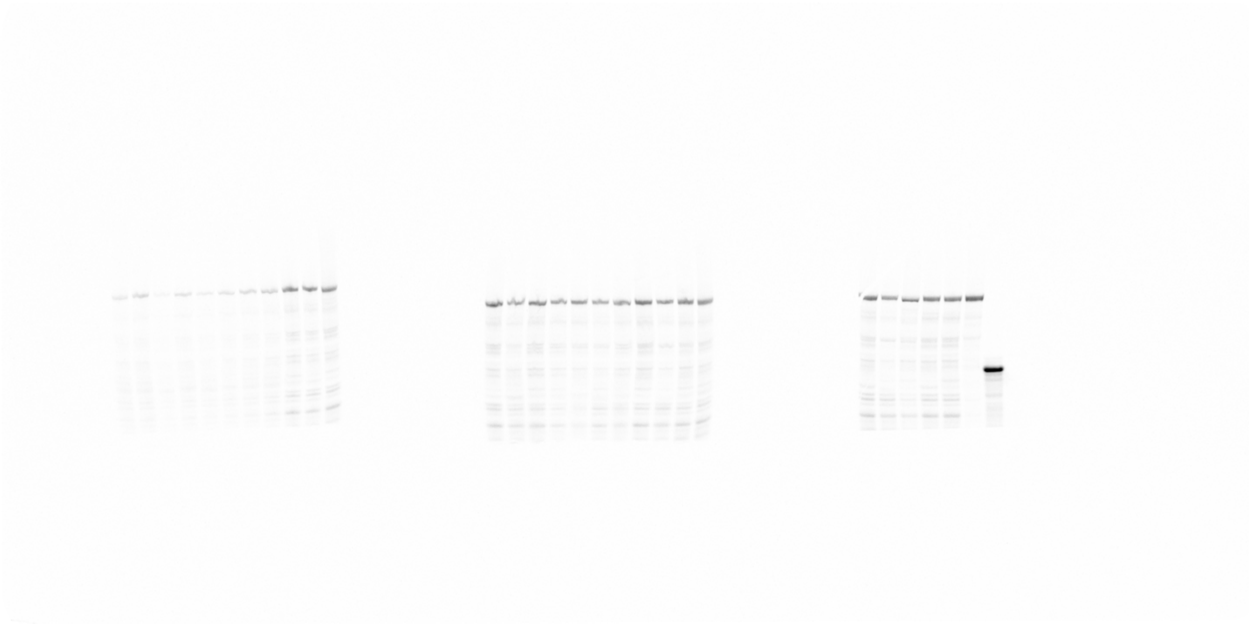

For Supplementary Figure 6:

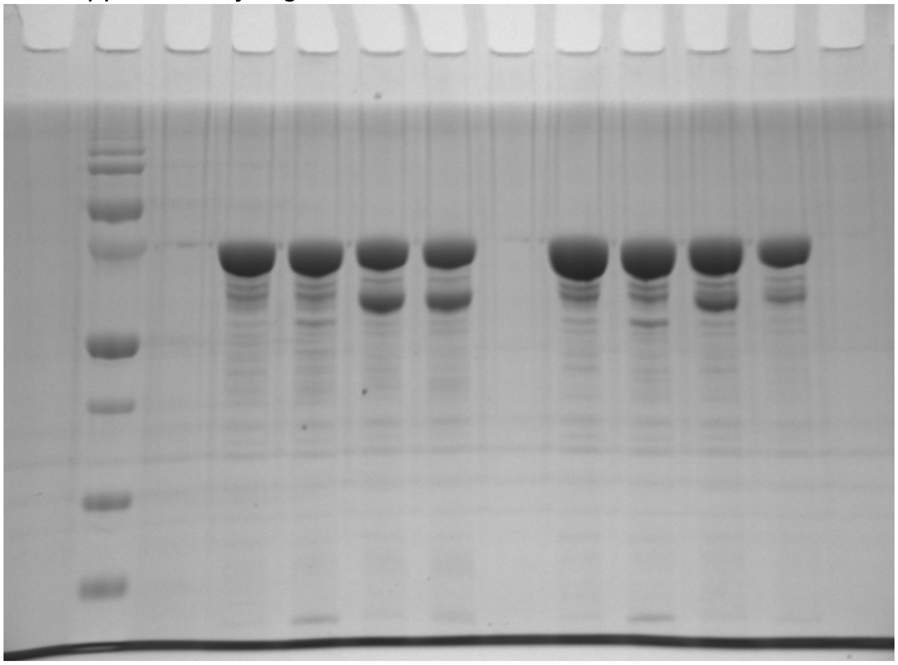

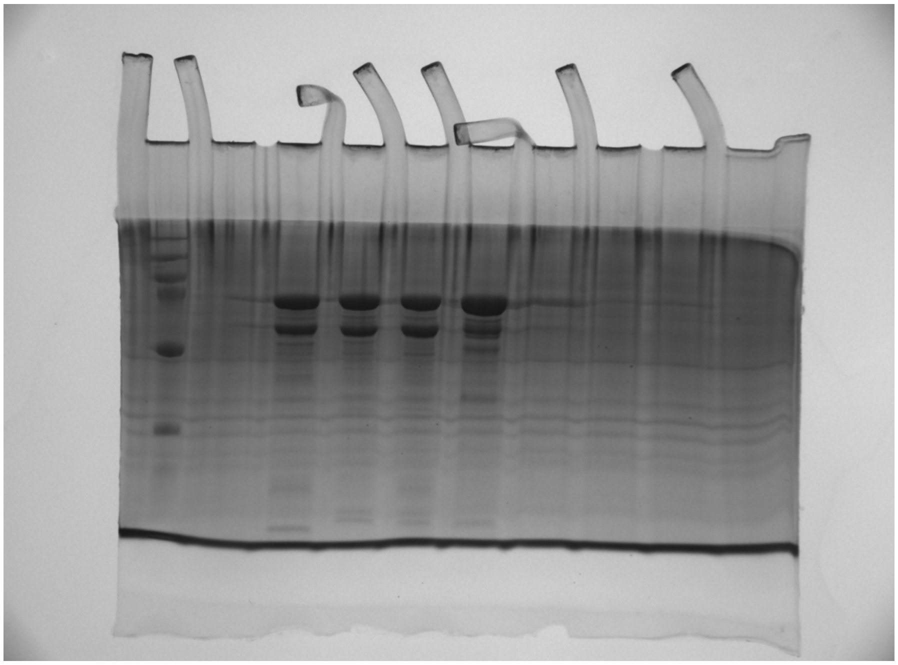

For Supplementary Figure 13:

2TAGsfGFP

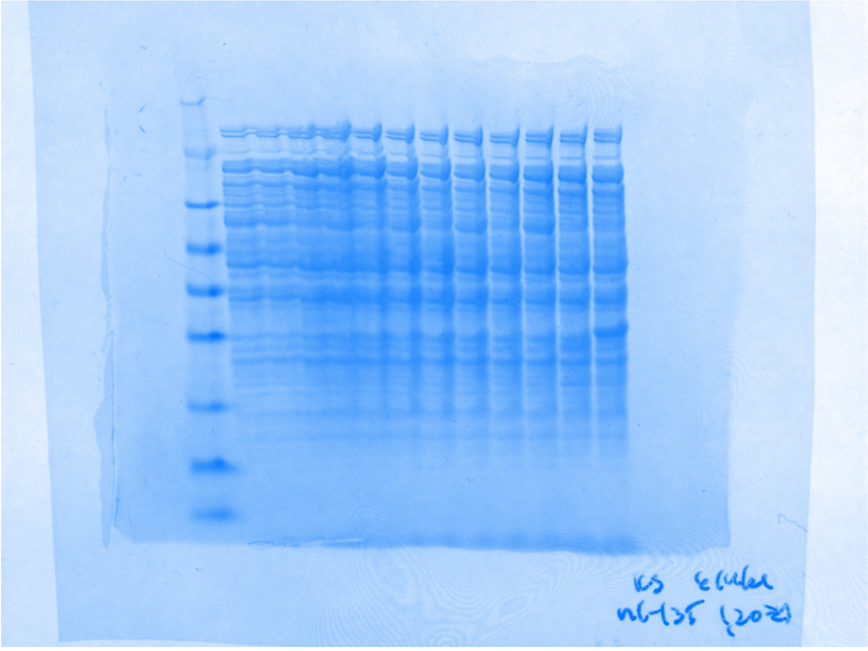

108.508

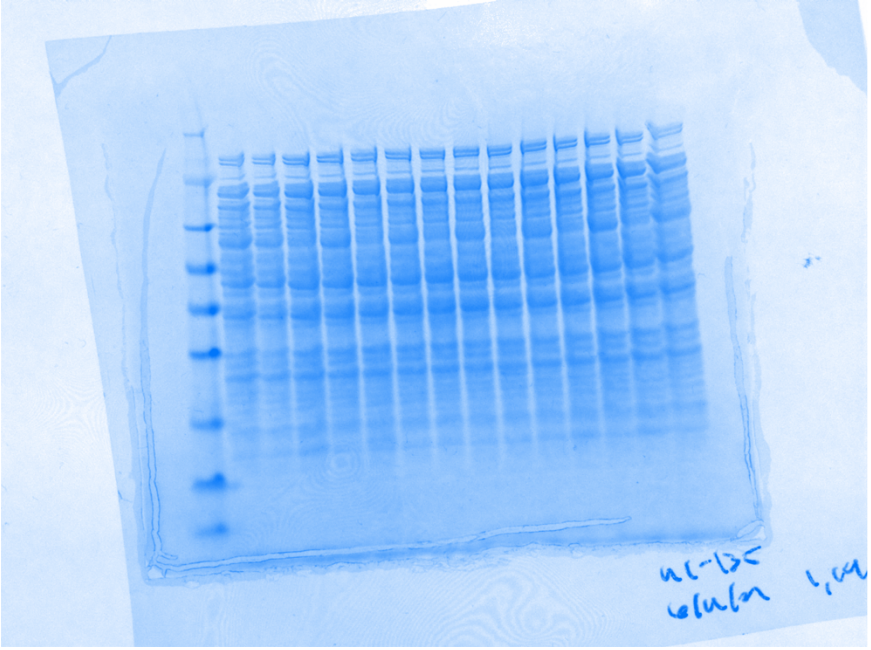

202.407

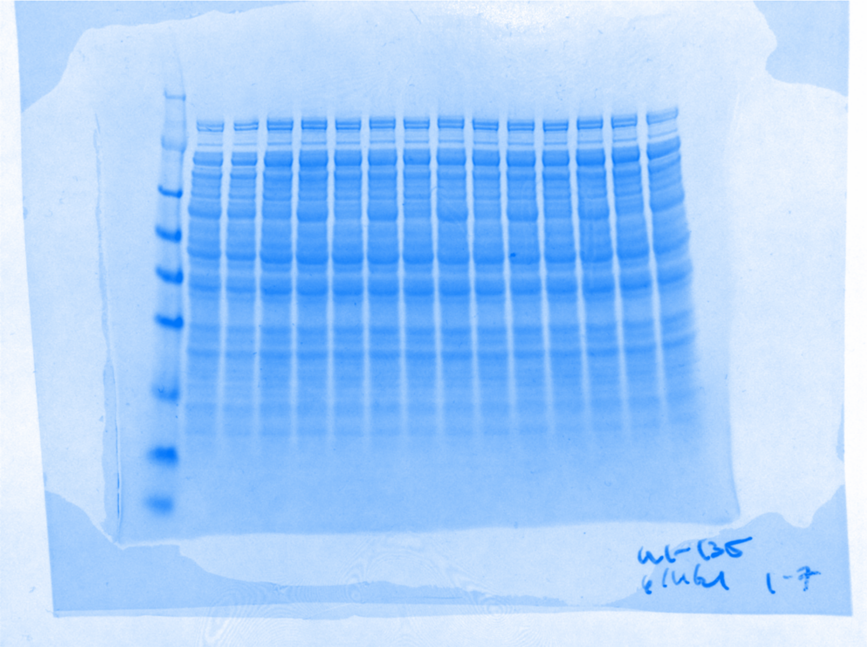

507.110

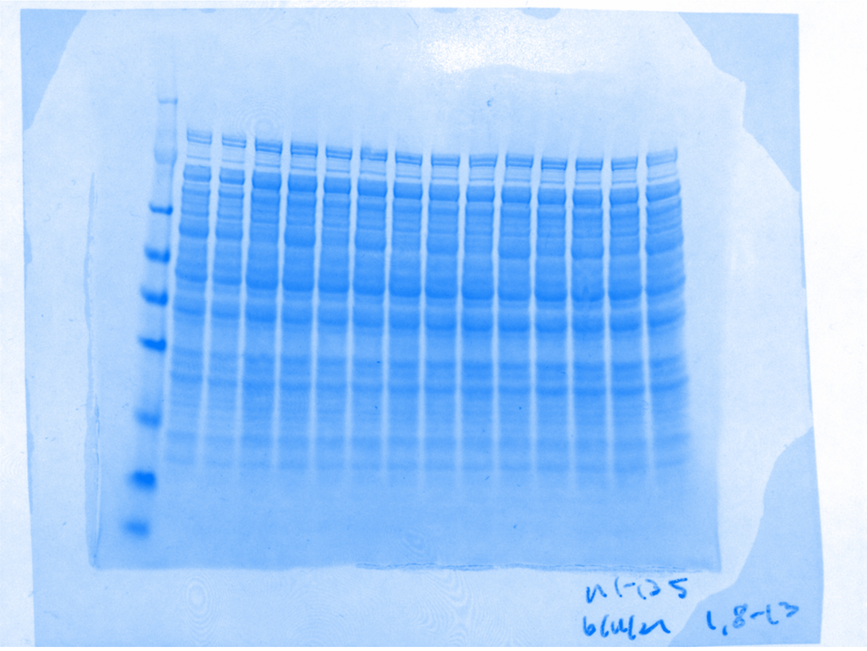

13b Uncropped Autoradiogram.tif

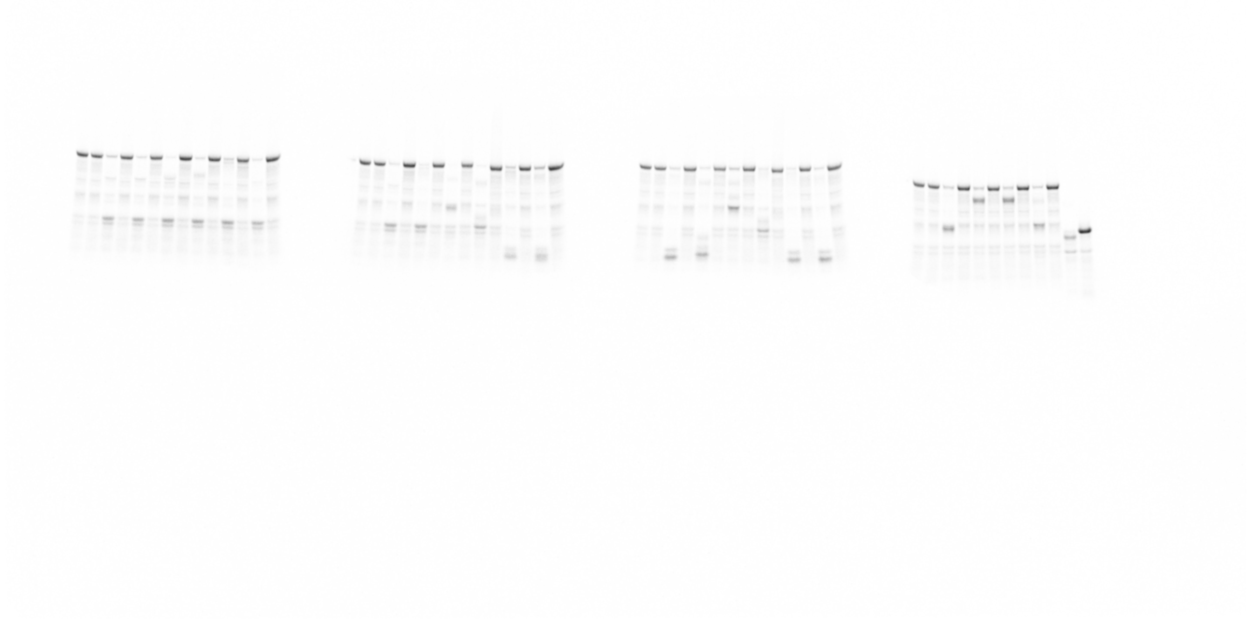

References

1. K, Y., K, I. & Y, N. Amber (UAG) suppressors affected in UGA/UAA-specific polypeptide release factor 2 of bacteria: genetic prediction of initial binding to ribosome preceding stop codon recognition. *Genes Cells* **4,** 253–266 (1999).
2. Mills, J. H. *et al.* Computational design of a homotrimeric metalloprotein with a trisbipyridyl core. *PNAS.* **113,** 15012–15017 (2016).
3. Meyer, T. J. Photochemistry Of Metal Coordination Complexes: Metal To Ligand Charge Transfer Excited States. *Pure Appl. Chem.* **58,** 1193–1206 (1986).
4. Zernickel, A. V. Artificial Metalloenzymes through Chemical Modification of Engineered Host Proteins. (King Abdullah University of Science and Technology, 2014).
5. Polin, J., Schmohel, E. & Balzani, V. Functionalization of 2,2′-Bipyridines in Their 4 and 5 Positions. Synthesis of 5-Ethynyl-2,2′-bipyridine. *Synthesis* **3,** 321–324 (1998).
6. Ballardini, R. *et al.* Photoinduced Electron Transfer in a Triad That Can Be Assembled/Disassembled by Two Different External Inputs. Toward Molecular-Level Electrical Extension Cables. *JACS* **124,** 12786–12795 (2002).
7. Xie, J., Liu, W. & Schultz, P. G. A Genetically Encoded Bidentate, Metal-Binding Amino Acid. *Angew. Chemie Int. Ed.* **46,** 9239–9242 (2007).
8. Park, N., Ryu, J., Jang, S. & Lee, H. S. Metal ion affinity purification of proteins by genetically incorporating metal-chelating amino acids. *Tetrahedron* **68,** 4649–4654 (2012).
9. Roux, B. The calculation of the potential of mean force using computer simulations. *Comput. Phys. Commun.* **91,** 275–282 (1995).
10. Yoo, J. & Aksimentiev, A. Refined Parameterization of Nonbonded Interactions Improves Conformational Sampling and Kinetics of Protein Folding Simulations. *J. Phys. Chem. Lett.* **7,** 3812–3818 (2016).
11. Smith, R. M. & Martell, A. E. *Critical Stability Constants Volume 2: Amines* 235-237 (Plenum Press, New York, 1975).
12. Roux, B. & Simonson, T. Implicit solvent models. *Biophys. Chem.* **78,** 1–20 (1999).

13. Won, Y. Force Field for Monovalent, Divalent, and Trivalent Cations Developed under the Solvent Boundary Potential. *J. Phys. Chem. A* **116,** 11763–11767 (2012).
14. Humphrey, W., Dalke, A. & Schulten, K. VMD: Visual molecular dynamics. *J. Mol. Graph.* **14,** 33–38 (1996).
15. S, J., T, K., VG, I. & W, I. CHARMM-GUI: a web-based graphical user interface for CHARMM. *J. Comput. Chem.* **29,** 1859–1865 (2008).
16. Phillips, J. C. *et al.* Scalable molecular dynamics on CPU and GPU architectures with NAMD. *J. Chem. Phys.* **153,** 044130 (2020).
17. Best, R. B. *et al.* Optimization of the Additive CHARMM All-Atom Protein Force Field Targeting Improved Sampling of the Backbone ϕ, ψ and Side-Chain χ1 and χ2 Dihedral Angles. *J. Chem. Theory Comput.* **8,** 3257–3273 (2012).
18. Jorgensen, W. L., Chandrasekhar, J., Madura, J. D., Impey, R. W. & Klein, M. L. Comparison of simple potential functions for simulating liquid water. *J. Chem. Phys.* **79,** 926 (1998).
19. Klauda, J. B. *et al.* Update of the CHARMM All-Atom Additive Force Field for Lipids: Validation on Six Lipid Types. *J. Phys. Chem. B* **114,** 7830–7843 (2010).
20. A. D. MacKerell, J. *et al.* All-Atom Empirical Potential for Molecular Modeling and Dynamics Studies of Proteins †. *J. Phys. Chem. B* **102,** 3586–3616 (1998).
21. Vanommeslaeghe, K. *et al.* CHARMM general force field: A force field for drug-like molecules compatible with the CHARMM all-atom additive biological force fields. *J. Comput. Chem.* **31,** 671–690 (2010).
22. Sundlov, J. A., Fontaine, D. M., Southworth, T. L., Branchini, B. R. & Gulick, A. M. Crystal Structure of Firefly Luciferase in a Second Catalytic Conformation Supports a Domain Alternation Mechanism. *Biochemistry* **51,** 6493–6495 (2012).
23. Ellis-Guardiola, K. *et al.* Crystal Structure and Conformational Dynamics of *Pyrococcus furiosus* Prolyl Oligopeptidase. *Biochemistry* **58,** 1616–1626 (2019).
24. Kightlinger, W. *et al.* Design of glycosylation sites by rapid synthesis and analysis of glycosyltransferases article. *Nat. Chem. Biol.* **14,** 627–635 (2018).
25. Kwon, Y. C. & Jewett, M. C. High-throughput preparation methods of crude extract for robust cell-free protein synthesis. *Sci. Rep.* **5,** 8663 (2015).
26. Jewett, M. C. & Swartz, J. R. Rapid Expression and Purification of 100 nmol Quantities of Active Protein Using Cell-Free Protein Synthesis. *Biotechnol. Prog.* **20,** 102–109 (2004).
27. Harris, M. N., Madura, J. D., Ming, L. J. & Harwood, V. J. Kinetic and Mechanistic Studies of Prolyl Oligopeptidase from the Hyperthermophile Pyrococcus furiosus. *J. Biol. Chem.* **276,** 19130-19137 (2001).
28. Srivastava, P., Yang, H., Ellis-Guardiola, K. & Lewis, J. C. Engineering a dirhodium artificial metalloenzyme for selective olefin cyclopropanation. *Nat. Commun.* **6,** 7789 (2015).
