# Supplementary material for: Metal-responsive regulation of enzyme catalysis using genetically encoded chemical switches
Source: Nat Commun. 2022 Apr 6;13:1864. doi: 10.1038/s41467-022-29239-y (PMC8987029; doi:10.1038/s41467-022-29239-y)
Supplement: Supplementary file 2 — Reporting Summary [file 41467_2022_29239_MOESM2_ESM.pdf]

nature portfolio

Corresponding author(s): Jared Lewis

Last updated by author(s): March 2 2022

Reporting Summary

Nature Portfolio wishes to improve the reproducibility of the work that we publish. This form provides structure for consistency and transparency in reporting. For further information on Nature Portfolio policies, see our Editorial Policies and the Editorial Policy Checklist.

Statistics

For all statistical analyses, confirm that the following items are present in the figure legend, table legend, main text, or Methods section.

n/a	Confirmed	
☐	☒	The exact sample size (n) for each experimental group/condition, given as a discrete number and unit of measurement
☐	☒	A statement on whether measurements were taken from distinct samples or whether the same sample was measured repeatedly
☒	☐	The statistical test(s) used AND whether they are one- or two-sided<br>*Only common tests should be described solely by name; describe more complex techniques in the Methods section.*
☒	☐	A description of all covariates tested
☒	☐	A description of any assumptions or corrections, such as tests of normality and adjustment for multiple comparisons
☐	☒	A full description of the statistical parameters including central tendency (e.g. means) or other basic estimates (e.g. regression coefficient) AND variation (e.g. standard deviation) or associated estimates of uncertainty (e.g. confidence intervals)
☒	☐	For null hypothesis testing, the test statistic (e.g. F, t, r) with confidence intervals, effect sizes, degrees of freedom and P value noted<br>*Give P values as exact values whenever suitable.*
☒	☐	For Bayesian analysis, information on the choice of priors and Markov chain Monte Carlo settings
☒	☐	For hierarchical and complex designs, identification of the appropriate level for tests and full reporting of outcomes
☒	☐	Estimates of effect sizes (e.g. Cohen's d, Pearson's r), indicating how they were calculated

Our web collection on statistics for biologists contains articles on many of the points above.

Software and code

Policy information about availability of computer code

Data collection	Protein MS were collected with Waters MassLynx 4.1; UV-Vis data for Michaelis-Menten kinetics and POP switching assays were collected with Cary WinUV; UV-Vis spectra for POP variants were collected with Cary WinUV2; CD spectra for POP variants were collected with JASCO Spectra Manager Spectra Measurement; Gen5 Version 2.09.2, Build 2.09.1 was used for plate readers and enzyme kinetics measurements; Typhoon FLA7000 Version 1.2, Build 1.2.1.93 was used for imaging autoradiograms; Echo Plate Reformat Version 1.7.2, Build 24 was used to set up POP enzyme reactions; Microbeta2, Version 1.0 Sp1 was used for scintillation counting for quantification of proteins within CFPS.
Data analysis	Data analysis was conducted using Excel Version 2108, OriginPro 2021 (64-bit) 9.8.0.200 (Academic) , PyMOL 2.5.0, Compass Data Analysis 5.1 Build 201.2.4019 (Bruker) for MS data, and Python 3.7 using custom scripts. Codes have been deposited in Zenodo as requested under DOI: 10.5281/zenodo.6320869. This is mentioned in the manuscript

For manuscripts utilizing custom algorithms or software that are central to the research but not yet described in published literature, software must be made available to editors and reviewers. We strongly encourage code deposition in a community repository (e.g. GitHub). See the Nature Portfolio guidelines for submitting code & software for further information.

Data

Policy information about availability of data

All manuscripts must include a data availability statement. This statement should provide the following information, where applicable:

- Accession codes, unique identifiers, or web links for publicly available datasets
- A description of any restrictions on data availability
- For clinical datasets or third party data, please ensure that the statement adheres to our policy

The data used to generate Figures 2-5 are provided in the Source Data file. Complete experimental methods, characterization, and supplementary data/figures are available in the Supplementary Information. POP structure 5T88 was obtained from the Protein Data Bank.

Field-specific reporting

Please select the one below that is the best fit for your research. If you are not sure, read the appropriate sections before making your selection.

- [x] Life sciences
- [ ] Behavioural & social sciences
- [ ] Ecological, evolutionary & environmental sciences

For a reference copy of the document with all sections, see nature.com/documents/nr-reporting-summary-flat.pdf

Life sciences study design

All studies must disclose on these points even when the disclosure is negative.

Sample size	Reactions for kinetic analysis of enzymes were performed in triplicate throughout this study. One replicate of physical spectroscopic characterization of POP variants (e.g. UV-Vis, CD, MS) and luciferase variants (e.g. MS) was performed. Audiogram experiments were also performed once.
Data exclusions	No data were excluded from analysis
Replication	Both screening and validation kinetic assays were performed with three technical replicates. Furthermore, the screening and validation assays were performed in different labs by different researchers, and results were similar, demonstrating that data from key experiments can be replicated. All attempts to replicate were successful. All experiments were repeated at least once and the data presented are representative of one of those experiments.
Randomization	There were no experimental groups in this study
Blinding	There was no blinding needed in this study because multiple authors obtained similar results for replicates.

Reporting for specific materials, systems and methods

We require information from authors about some types of materials, experimental systems and methods used in many studies. Here, indicate whether each material, system or method listed is relevant to your study. If you are not sure if a list item applies to your research, read the appropriate section before selecting a response.

Materials & experimental systems

n/a	Involved in the study	
x		Antibodies
x		Eukaryotic cell lines
x		Palaeontology and archaeology
x		Animals and other organisms
x		Human research participants
x		Clinical data
x		Dual use research of concern

Methods

n/a	Involved in the study	
x		ChIP-seq
x		Flow cytometry
x		MRI-based neuroimaging
